# Blind method for discovering number of clusters in multidimensional datasets by regression on linkage hierarchies generated from random data

**DOI:** 10.1371/journal.pone.0227788

**Published:** 2020-01-23

**Authors:** Osbert C. Zalay

**Affiliations:** Division of Radiation Oncology, Department of Oncology, Queen’s University, Kingston, Ontario, Canada; Beijing University of Technology, CHINA

## Abstract

Determining intrinsic number of clusters in a multidimensional dataset is a commonly encountered problem in exploratory data analysis. Unsupervised clustering algorithms often rely on specification of cluster number as an input parameter. However, this is typically not known *a priori*. Many methods have been proposed to estimate cluster number, including statistical and information-theoretic approaches such as the gap statistic, but these methods are not always reliable when applied to non-normally distributed datasets containing outliers or noise. In this study, I propose a novel method called *hierarchical linkage regression*, which uses regression to estimate the intrinsic number of clusters in a multidimensional dataset. The method operates on the hypothesis that the organization of data into clusters can be inferred from the hierarchy generated by partitioning the dataset, and therefore does not directly depend on the specific values of the data or their distribution, but on their relative ranking within the partitioned set. Moreover, the technique does not require empirical data to train on, but can use synthetic data generated from random distributions to fit regression coefficients. The trained hierarchical linkage regression model is able to infer cluster number in test datasets of varying complexity and differing distributions, for image, text and numeric data, using the same regression model without retraining. The method performs favourably against other cluster number estimation techniques, and is also robust to parameter changes, as demonstrated by sensitivity analysis. The apparent robustness and generalizability of hierarchical linkage regression make it a promising tool for unsupervised exploratory data analysis and discovery.

## Introduction

Clustering is an essential tool for data discovery, and automated clustering techniques constitute a major branch of unsupervised learning. However, there is a fundamental challenge that arises anytime a clustering algorithm is applied: namely, determining the intrinsic number of clusters present. Most clustering algorithms take cluster number either as an implicit or explicit model parameter; yet, the actual number of groupings present within a dataset is often not known *a priori*.

There are various methods available to estimate cluster number when it is not known. These include basic empirical approaches such as data visualization or simple trial-and-error, and more sophisticated statistical or information-theoretic methods, for example, optimizing some measure of internal or inter-cluster variability or validity [1–6]. There also exist automated or semi-automated clustering algorithms that can implicitly discover cluster number, or at minimum, iteratively deduce a clustering solution when given a set of prototype clusters or seed points, of which some examples include network clustering [7], projection and/or ensemble clustering [8], density-based clustering [9,10], and probabilistic clustering [11], as well as extensions to conventional clustering methods such as k-means using optimization techniques [12]. However, many of these methods are heuristic, or contain heuristic elements, so that there are situations in which they work well and others in which they do not, without definition of formal conditions guaranteeing an optimal solution.

In this paper, I present a new technique called *hierarchical linkage regression* (HLR), which is a blind method for inferring the number of clusters in an unknown dataset through regression on linkage hierarchies, which are utilized in hierarchical clustering [13]. Although regression does not guarantee an optimal solution, once a regression model is trained, the behaviour of the regression tends to be robust, and it is possible to establish confidence bounds on the uncertainty of the estimate. To define the regression problem, the underlying hypothesis is that the hierarchical organization of data points in a multidimensional feature space is informative of their natural clustering pattern, independent of the data values themselves or the magnitude of the separation between data points (i.e. it should not depend on how the data are uniquely distributed). If this holds true, then regression on linkage hierarchies derived from mixtures of random clusters of synthetic data can be used to infer the number of clusters in an experimental dataset that the model has not seen before. The practical implementation relies on the fact that there are limited number of ways to partition finite data, so that predictable patterns emerge with respect to how data of a specific cluster number are organized. Therefore, it becomes possible to extract information about intrinsic clustering properties by generating many different instances of clustered data for the model to learn from, so that the regression coefficients can be determined through an iterative optimization process.

To test the hypothesis and demonstrate practical implementation of the technique, a small neural network was trained to perform hierarchical linkage regression to estimate actual number of clusters in real-world data from image, text and numeric formats. Image data were sourced from standardized texture databases grouped according to texture classes of sample materials photographed under different angles, scales and lighting conditions [14–16]. Text data were obtained from Wikipedia and words grouped by degree of semantic similarity, as determined by distances between vectorized word embeddings [17]. Several other real-world datasets were tested as well, specifically the iris [18], wine [19], glass [20], and spam datasets [21], which were also datasets analysed by Flexa et al. using their mutual equidistant-scattering criterion for clustering, against which they compared a range of different clustering techniques [6]. In this paper, the performance of HLR for cluster number estimation on image and text data was compared head-to-head against statistical or information-theoretic techniques, including the gap statistic [5], silhouette method [3], the variance ratio criterion (Calinski-Harabasz index) [1], and the Davies-Bouldin criterion [2], as well as the modern automated clustering techniques of network clustering (affinity propagation [7]) and density clustering (DBSCAN [10]and OPTICS [9]). HLR was found to be more robust compared with both standard and automated techniques when tested on text and image data. Sensitivity analysis of HLR model parameters was also carried out by comparing cluster estimation accuracy and uncertainty for different values of the model parameters, thereby providing guidance on appropriate parameter selection for the proposed method.

## Materials and methods

In this section, the methodology underlying hierarchical linkage regression will be presented, as well as the practical aspects of model implementation, including the data used for model training and evaluation.

### 2.1. Linkage analysis

Let there be an initial set (partition) of *M* individual data elements with *n* dimensions each:

$$P_{M}=\{x_{1},x_{2},\ldots ,x_{M}\},\;x\in R^{n}$$ (1)

where the *i*th element can also be viewed as a solitary vector belonging to its own subset, *X*_*i*_ = {***x***_*i*_}, *i* = 1, …, *M*. Now define an element-wise distance function, *d*(*X*_*i*_, *X*_*j*_) = *d*(***x***_*i*_, ***x***_*j*_), such that a linkage is made between elements satisfying the smallest pairwise distance between them:

$$x_{j}^{*},x_{k}^{*}=\underset{\forall j\neq k}{\text{argmin}}d(X_{i},X_{j}).$$ (2)

such that combination of $X_{j}^{*}=\{x_{j}^{*}\}$ and ${X_{k}^{*}=\{x}_{k}^{*}\}$ creates a new subset $X_{M+1}=\{x_{j}^{*},x_{k}^{*}\}$, thereby partitioning the original set, *P*_*M*_, into a new agglomerate set with *M* − 1 elements:

$$P_{M-1}=\{X_{1},\ldots X_{j-1}^{*},X_{j+1}^{*},\ldots X_{k-1}^{*},X_{k+1}^{*},\ldots ,X_{M},X_{M+1}\}.$$ (3)

This process is repeated another *M* − 2 times, combining pairs of elements with the smallest separation between them, until all pairwise groupings are exhausted, providing a hierarchical sequence of nested partitions *P*_*M* − 1_ …*P*_1_ for the entire dataset.

**Fig 1:**
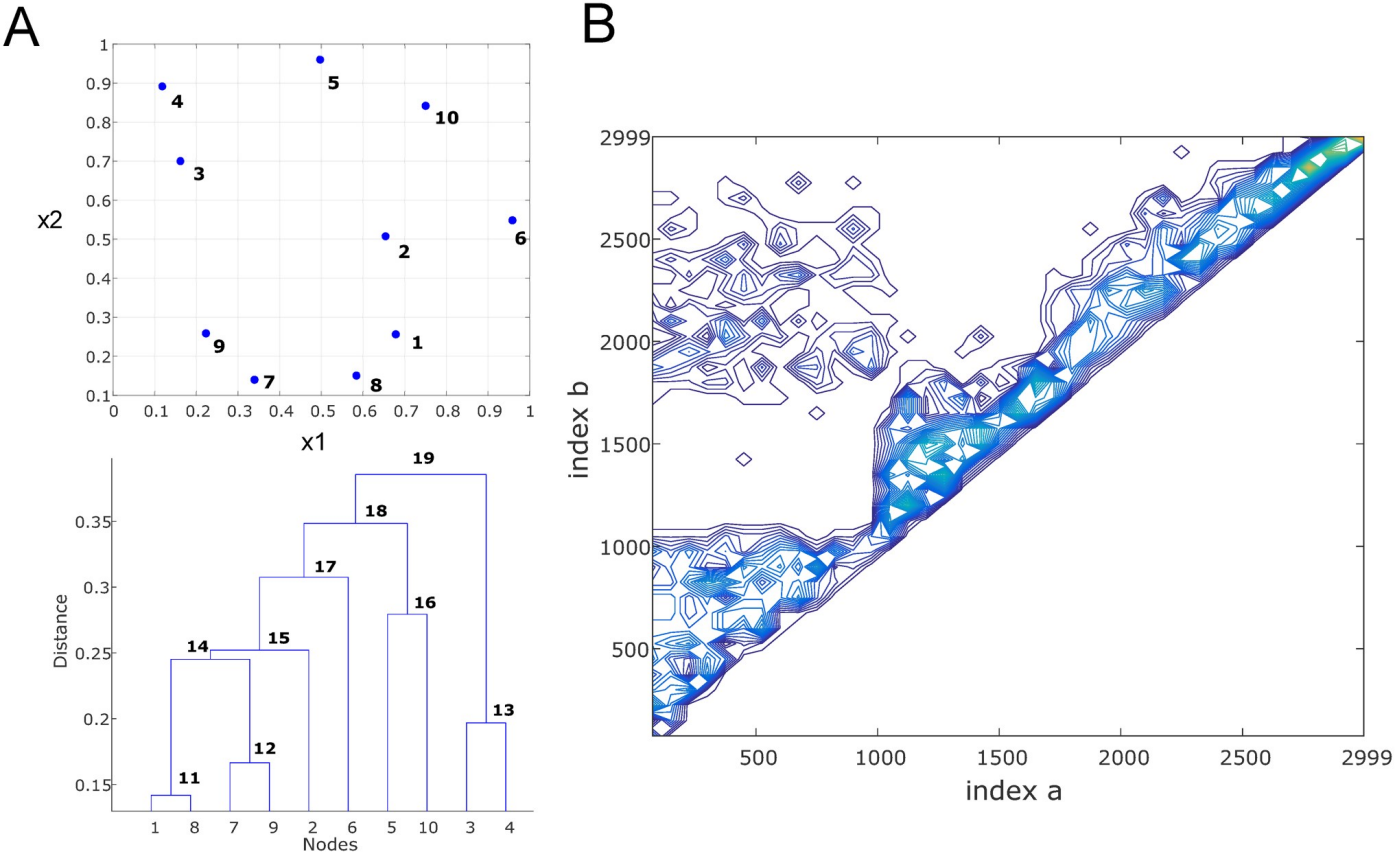
Illustration of linkage analysis. (A) Example of *M* = 10 data points randomly placed on 2-dimensional coordinate plane (x1, x2), and resultant linkage analysis using single link distance function with Euclidean (L2 norm) distance metric. The leaf nodes of the dendrogram (lower plot), numbered 1 to 10, represent the original data points, and the branch nodes, numbered from *M* + 1 to 2*M –* 1 represent the pairwise linkages based on separation hierarchy. (B) Contour plot of 2-dimensional histogram of linkage coordinate indices (*a*, *b*) corresponding to the linkage hierarchy for a randomly generated pair of clusters formed from *M* = 1500 data points (500 for cluster #1 and 1000 for cluster #2). Due to index condition *b > a*, the values below the diagonal are zero.

The pairwise hierarchy of partitions is vertically organized as a binary tree (a dendrogram), such that the original points in the dataset are represented by *M* leaf nodes of the tree, and the *M* − 1 levels above are the branch nodes, each level representing a single pairing between two elements in the set (Fig 1A). Pairings low down on the tree are closer in separation than those further up the tree, providing information on how the data points are organized within the partitioning hierarchy. This information is encoded by the branch nodal indices of the binary tree, whereby the higher the index value, the higher up the level in the tree. Therefore, a set of coordinates, L, made up of hierarchical linkage indices can be specified, such that

$$\begin{array}{c} L=\left\{{\left(a_{1},b_{1}\right)}_{X_{M+1}},{\left(a_{2},b_{2}\right)}_{X_{M+2}},\ldots ,{\left(a_{M-1},b_{M-1}\right)}_{X_{M+\left(M-1\right)}}\right\},\\ 1\leq a<b<2M-1 \end{array}$$ (4)

where the coordinates $\lambda_{i}={\left(a_{i},b_{i}\right)}_{x_{M+i}}$ of the *i*th level pairing, whose linkage is assigned the index value *M* + *i*, is comprised of the coordinate indices *a*_*i*_ and *b*_*i*_ of the lower-level linkages that formed it.

To obtain a feature vector from the indices, a 2-dimensional histogram of the linkage coordinates can be generated (Fig 1B) wherein the *a*-axis and *b*-axis of the coordinate indices are subdivided into *N*_*a*_ intervals and *N*_*b*_ intervals, respectively, forming a *N*_*a*_ × *N*_*b*_ rectangular grid in which the coordinate pairings are binned and counted, thus capturing statistical information about the distribution of the pairings that preserves their hierarchical relationship:

$$h_{j,k}=\frac{1}{M-1}\sum_{i=1}^{M-1}\delta_{j,k}(\lambda_{i})$$ (5)

where the discrete Kronecker delta is defined by values of the coordinate indices from Eq (4),

$$\begin{array}{c} \delta_{j,k}\left(\lambda_{i}\right)=\{\begin{array}{cc} 1, & j\varepsilon_{a}<a_{i}\leq \left(j+1\right)\varepsilon_{a},\;k\varepsilon_{b}<b_{i}\leq \left(k+1\right)\varepsilon_{b}\\ 0, & otherwise \end{array}\\ j=0,1,\ldots ,N_{a}-1\\ k=0,1,\ldots ,N_{b}-1 \end{array}$$ (6)

and

$$\varepsilon_{r}=\frac{2M-1}{N_{r}},\;r=\{a,b\}$$ (7)

is the length of the side of the bin formed by subdividing the *r*-axis into *N*_*r*_ equal intervals. The histogram is normalized by *M* − 1 so that bin values fall in the range 0 to 1, and “unrolled” to obtain a feature vector of length *N*_*a*_ × *N*_*b*_. However, at least half of the values of the histogram are zero (due to the index condition *b* > *a*), so the feature vector can be reduced by excluding elements of the histogram below the matrix diagonal which are always zero. For purposes of the analysis in this paper, I set the bin numbers equal to each other, *N*_*a*_ = *N*_*b*_ = *R*, resulting in an unrolled feature vector of reduced dimension *R*(*R* + 1)/2.

### 2.2 Distance functions

Although the linkage histogram and feature vectors are not directly dependent on the magnitudes of the distances between data points, and are determined by linkage hierarchy, a distance metric is nonetheless required to construct the hierarchy. In this paper, the L1 norm (Manhattan distance) was compared to the more ubiquitous L2 norm (Euclidean distance) for purposes of linkage analysis, as better performance tends to be observed for L*k* norms with *k* ≤ 2 in higher-dimensional spaces [22].

Element-wise distance functions that compute the distance between two sets as a function of their constituent elements have a more specialized form incorporating the aforementioned distance metrics. For this paper, single linkage, complete linkage and Ward’s method were evaluated for computing element-wise distances [23]. The single link distance function takes the form

$$d(X_{m},X_{n})=\underset{\begin{array}{c} x_{i}\in X_{m}\\ x_{j}\in X_{n} \end{array}}{\text{min}}{∥x_{i}-x_{j}∥}_{k}$$ (8)

where *k* = {1, 2} represents either the Manhattan or Euclidean norm, respectively. Therefore, the single link distance is defined as the shortest distance between all possible pairings of elements in *X*_*m*_ and in *X*_*n*_. Conversely, the complete link distance function takes the form

$$d(X_{m},X_{n})=\underset{\begin{array}{c} x_{i}\in X_{m}\\ x_{j}\in X_{n} \end{array}}{\text{max}}{∥x_{i}-x_{j}∥}_{k}$$ (9)

such that the element-wise distance is defined by the maximum separation between pairs of elements in *X*_*m*_ and in *X*_*n*_. Lastly, Ward’s method has a distance function of the form,

$$d(X_{m},X_{n})=\sqrt{2\frac{N_{m}N_{n}}{N_{m}{+N}_{n}}}{∥{\hat{x}}_{m}-{\hat{x}}_{n}∥}_{2}$$ (10)

where

$${\hat{x}}_{u}=\frac{1}{N_{u}}\sum_{x_{j}\in X_{u}}x_{j}$$ (11)

is the centroid of *X*_*u*_, and *N*_*u*_ is the number of elements in *X*_*u*_. Here the distance metric is by definition the Euclidean norm ${∥w∥}_{2}=\sqrt{w^{T}w}$. Ward’s distance function gives a measure of the increase in within-set variance caused by the merger of two subsets. The goal of Ward’s linkage is therefore to merge subsets having the smallest Ward’s distance, hence yielding the lowest increase in within-set variance.

### 2.3 Training data

According to the hypothesis, linkage hierarchy reflects how data are clustered, independent of the specific values of the data or the absolute magnitude of distances between data points themselves. This implies that it should be possible to take randomly generated data and cluster it in various known ways, and to use the clustering patterns from those synthetic data to train a supervised learning model to identify the relationship between linkage hierarchy and cluster pattern in experimental data, therefore enabling inference of cluster properties in real data that the model has not seen before. To generate an *n*-dimensional training dataset, let $z\in R^{n}$ be a composite random variable such that

$$z_{i}^{\left[l\right]}=c_{i}+v_{i}^{\left[l\right]}$$ (12)

where $c_{i}\sim U(w_{1},w_{2})$ is uniformly distributed on some interval $[w_{1},w_{2}]\in R^{n}$, marking the centroid of the *i*th cluster, and $v_{i}^{\left[l\right]}\sim N(0,\sigma_{i})$ is normally distributed with mean of zero and variance $\sigma_{i}\in R^{n}$, although in practice $v_{i}^{\left[l\right]}$ could be drawn from any distribution that best models the data of interest. The number of elements forming each cluster was selected at random as well.

### 2.4 Regression on linkage hierarchies

The process of generating a total of *K* instances of clustered random data, each with known number of assorted clusters, yields the training dataset *Z* = = {*Z*_1_, *Z*_2_, …, *Z*_*K*_}, where

$$Z_{j}=\{{z_{j}}_{1}^{\left[1\right]},{z_{j}}_{1}^{\left[2\right]},\ldots {,z_{j}}_{1}^{\left[l_{1}\right]},{z_{j}}_{2}^{\left[1\right]},{z_{j}}_{2}^{\left[2\right]},\ldots {,z_{j}}_{2}^{\left[l_{2}\right]},{\ldots ,z_{j}}_{Nj}^{\left[1\right]},{z_{j}}_{Nj}^{\left[2\right]},\ldots {,z_{j}}_{Nj}^{\left[l_{Nj}\right]}\}$$ (13)

such that *l*_*i*_ is the number of data points in *i*th cluster of the *j*th-clustering instance (defined by the first subscript of ***z***) with *N*_*j*_ clusters. The respective linkage hierarchies for the training set, *L*^(*Z*)^ = {*L*_1_, *L*_2_, …, *L*_*K*_}, defined in Eq (4), are computed from the *K* clustering instances and used to construct a $\frac{R(R+1)}{2}\times K$ dimensional feature matrix, ***F***^(*Z*)^, from the unrolled non-zero (diagonal and above) values of the linkage histograms defined by Eq (5):

$$F^{\left(Z\right)}=unroll\{h_{1},\ldots ,h_{K}\}=\left[\begin{array}{ccc} f_{1,1} & \cdots & f_{1,K}\\ \vdots & \ddots & \vdots \\ f_{\frac{R(R+1)}{2},1} & \cdots & f_{\frac{R(R+1)}{2},K} \end{array}\right].$$ (14)

**Fig 2:**
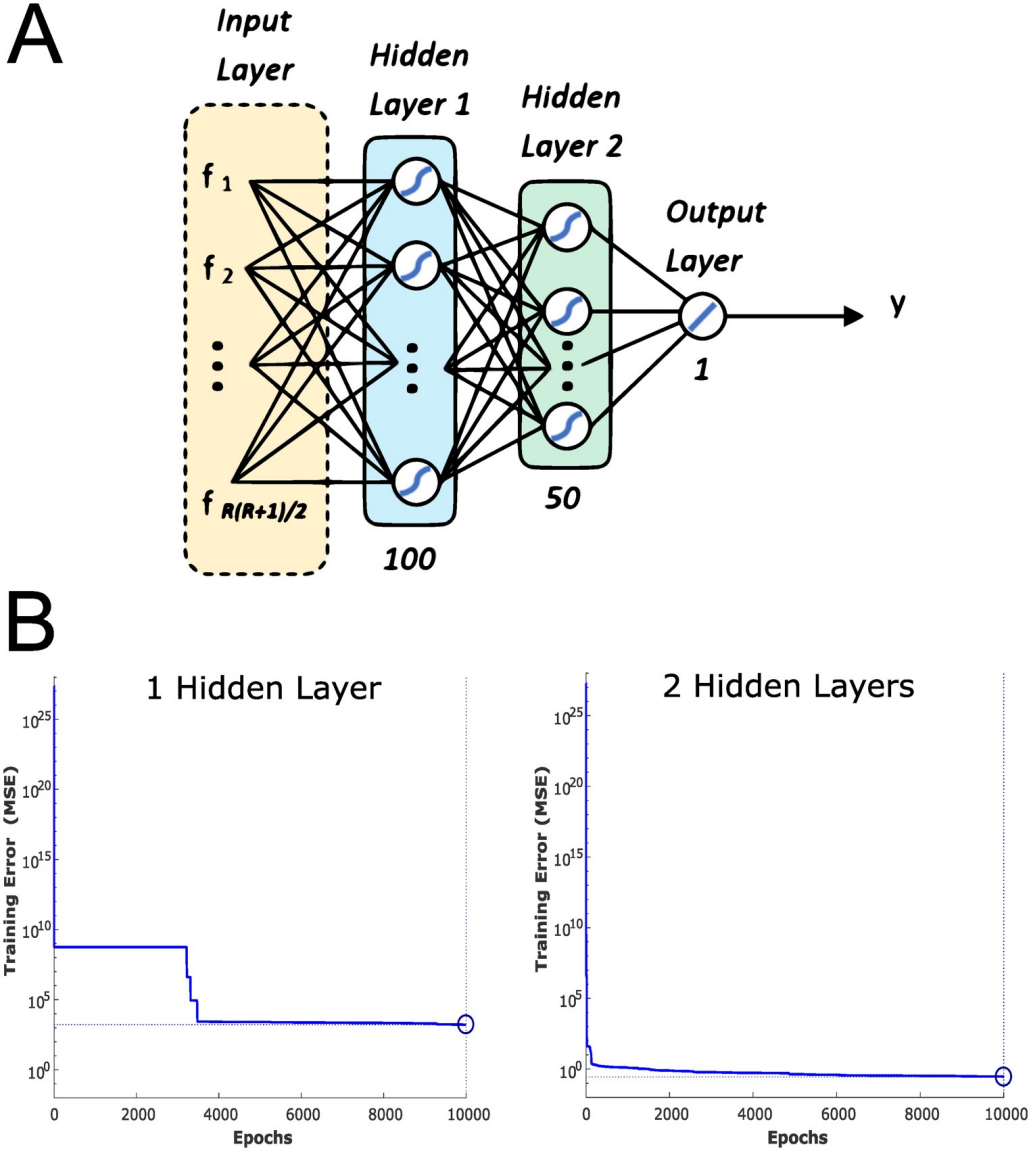
Design of neural network for hierarchical linkage regression. (A) Feedforward neural network architecture, with two hidden layers and single neuron output with linear activation. Inputs to the network are the unrolled, dimension-reduced feature vectors of the linkage coordinate histogram. (B) Effect of adding a second hidden layer to the neural network, resulting in more rapid convergence and lower training error. Solver: scaled conjugate gradient backpropagation (*β* = 0.2); hidden activations: sigmoid.

The columns of the feature matrix are then paired with the corresponding ground-truth cluster counts over the *K* instances,

$$\hat{y}={\left[{\hat{y}}_{1}\;{\hat{y}}_{2}\ldots {\hat{y}}_{K}\right]}^{T},\;\hat{y}\in \{I>0\}$$ (15)

and the relationship between linkage hierarchy and cluster number is discovered through regression on the training feature vectors:

$$y={g(F}^{\left(Z\right)},\Phi )$$ (16)

where **Φ** represents the combined matrix of coefficients (weights) learned by the regression model (the number of coefficients and hence dimensionality of the coefficient matrix depends on the structure of the model, *g*(⋅)). For this paper, a fully-connected feedforward neural network (FNN) was implemented to perform the regression. The FNN weights were learned using backpropagation with regularization, by scaled conjugate gradient method [24] unless otherwise specified, minimizing a mean squared error cost function of the form:

$$E(y,\Phi )=\frac{1}{K}{\left(y-\hat{y}\right)}^{T}(y-\hat{y})+\frac{\beta }{K}\sum_{i,j}{\phi_{i,j}}^{2}$$ (17)

where the regularization parameter, *β*, was adjusted within the range 0.2 to 0.8. The FNN was constructed with 2 hidden layers using sigmoid (or rectified linear) activation functions for all layers except the output layer, which consisted of a single neuron with linear activation (Fig 2A). Adding a second hidden layer improved convergence (Fig 2B). The trained model was then applied directly to the test datasets without cross-validation, because the purpose of this study was to test the hypothesis and demonstrate feasibility of the proposed method, and therefore it was not necessary to tune network hyperparameters to generate an optimal regression model. Software implementation for this paper was initially done in Matlab (Natick, MA), but a Python version has been coded that is available on Github for download [25].

### 2.5 Test data for model evaluation

#### 2.5.1 Image and text source data

Both image and text data were used to evaluate generalizability of the regression model, trained only on synthetic data, to various real-world data by performance comparison to other cluster number estimation techniques. Text data was sourced from Wikipedia. The test images were sourced from standardized texture databases, namely the Columbia-Utrecht Reflectance and Texture (CUReT) database [16], the University of Illinois Urbana-Champaign (UIUC) texture database [15] and the Swedish Royal Institute of Technology KTH-TIPS (Textures under varying Illumination, Pose and Scale) database [14]. These image databases make use of large variations in scale, resolution, lighting and orientation to increase classification difficulty, compared to the original Brodatz databases from which they take their inspiration. The CUReT database contains 61 different texture groups with 5612 images of 200 x 200 pixels. The UIUC database has 25 different grayscale textures with 1000 images of 640 x 480 pixels. The KTH-TIPS database has 10 grayscale texture classes comprising 810 images of 200 x 200 pixels. Features extracted from these three textural databases include multiscale local binary patterns (LBP) and Haralick features (HF) derived from grayscale co-occurrence matrices, which are well-established features for quantifying textures in images [26,27].

#### 2.5.2 Generating clustering instances

**Fig 3:**
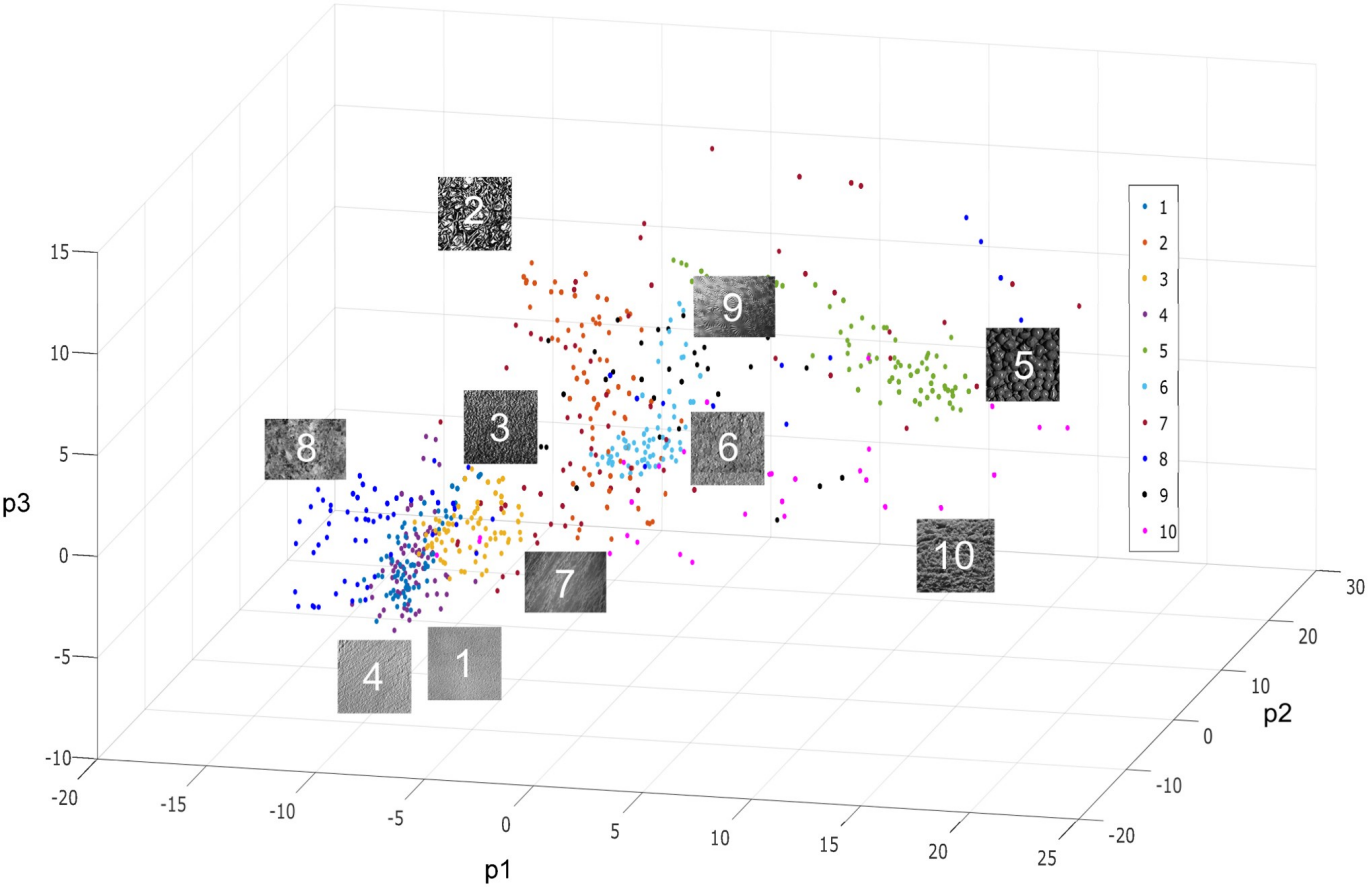
Example texture image data. This example has an assortment of 10 texture classes taken from the CuRET, UIUC and KTH-TIPS databases, each represented by a color-coded cluster, with each cluster consisting of a variable number of sample images taken under different orientations, lighting conditions and scales. Multi-resolution Haralick and local binary pattern texture features were computed, and then principal component analysis (PCA) was performed for visualization purposes and the data plotted with respect to the first three principal component axes. PCA was not performed as part of linkage analysis.

Generating multiple clustering instances was necessary for statistical comparison. For image data, the feature vectors were first computed for all of the images over all three databases, and then pooled into a single master set, with each texture class comprising a subset of feature vectors labelled with the same token. This was done to capture the heterogeneity of real-world data in testing the model. To generate a single instance of test data from the master set, a pseudo-random number generator was used to choose the following: (1) the number of clusters (i.e. texture classes) in a given instance; (2) the specific texture classes from which the image feature vectors would be sourced; and (3) and the number of samples (individual images) in each cluster, which was kept variable between clusters since real-world clusters in a dataset are not necessarily expected to be the same size. Finally, small noise perturbations were added to the data points so that clustering instances did not repeat exactly. The dimensionality of the test data was controlled by keeping the feature vector length as a parameter (more features could be generated by computing more LBP or HF values at different pixel radii). Fig 3 gives an example of clusters derived from the texture image data.

**Fig 4:**
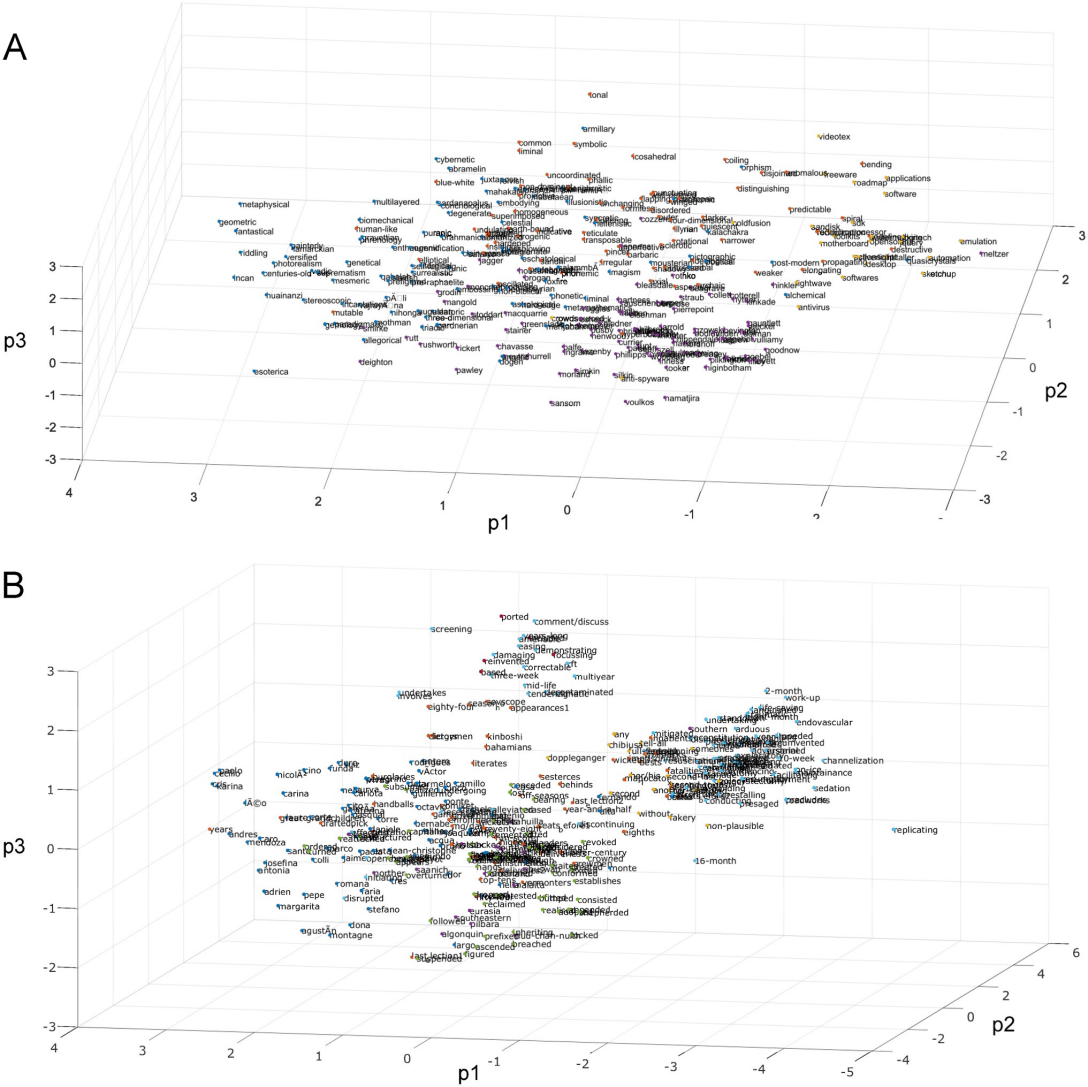
Example Wikipedia embedded text data. (A) Example of 4 clusters of Wikipedia words grouped by semantic similarity using word2vec embedding, plotting every second word for clarity of visualization. Principal component analysis (PCA) was used for data visualization (first three principal components were plotted), but was not used in linkage analysis. (B) Example of a 7-cluster set with every fifth word plotted.

For text data, the open-source Python library VSMlib was used to generate 25-dimensional word to vector (word2vec) embeddings from a pre-trained model using unbounded linear context continuous bag of words [28,29]. The source text was obtained from a 2013 Wikipedia English-language data dump, whereby the 14 GB text file was tokenized and parsed one word per line. The vectorized word embeddings could then be clustered according to semantic similarity based upon the distances between the nearest neighbouring word embeddings in 25-dimensional space. Similarly to the way clustered test data was generated from the image features, a single instance of the clustered text data was created by using a random number generator to select the following: (1) the number of clusters for the given instance; (2) a set of corresponding random words chosen from the source Wikipedia text, with minimum word length of 5 alpha-numeric letters or longer (this was to avoid biasing the test data with very common words like “is” or “the”), with each word designating a cluster seed point; and (3) the number of samples in each cluster, marking the nearest-neighbour word vectors to each randomly-selected seed word. Error checking was limited to eliminating repeated words when designating cluster seeds, but no minimum distance was set on how close cluster seeds were allowed to be, leaving to chance the degree of cluster overlap, which could be significant. Fig 4 illustrates examples of clustered word data from Wikipedia.

#### 2.5.3 How training and test data are distributed

**Fig 5:**
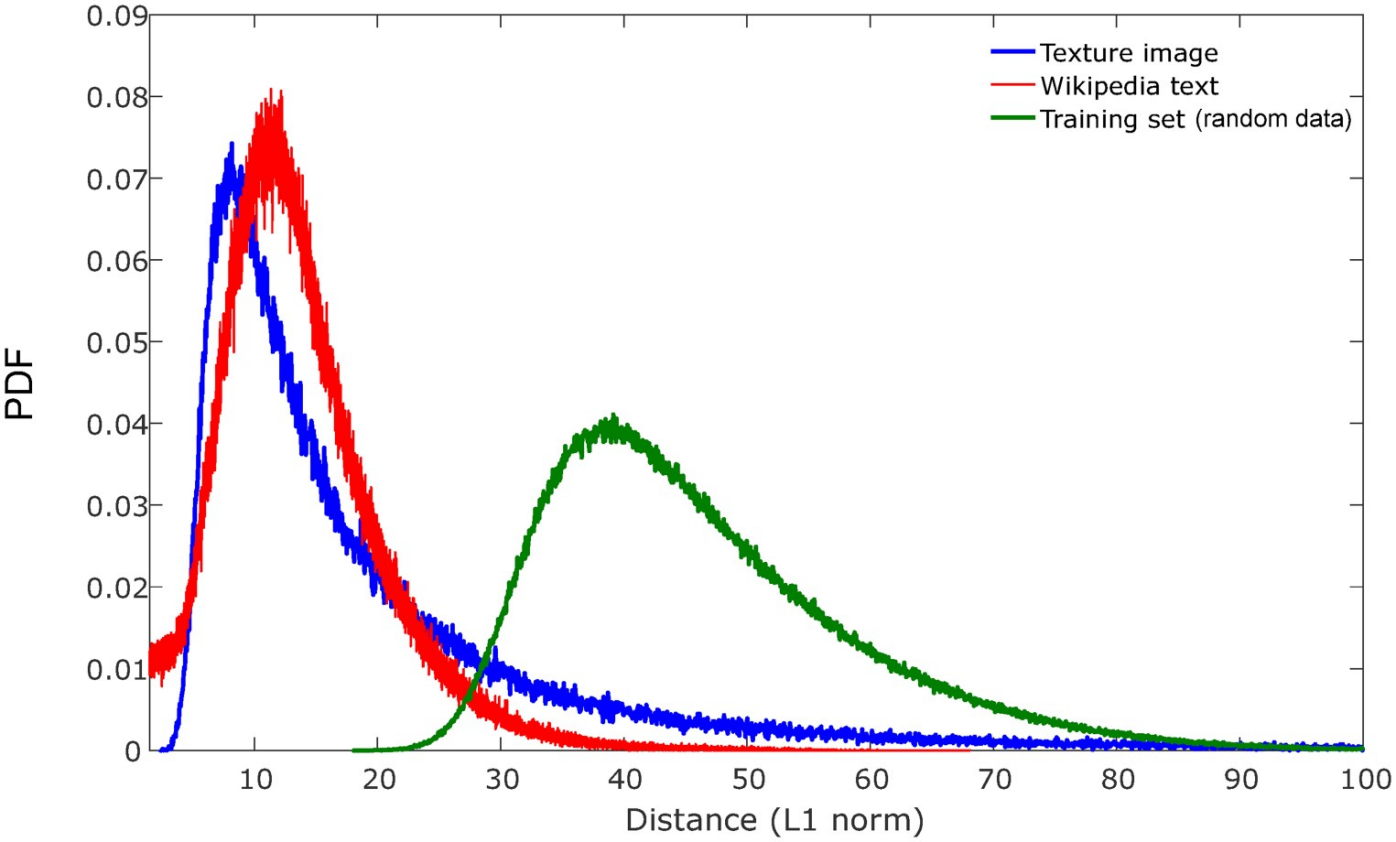
Aggregate distance (L1 norm) distributions of training, texture image and Wiki text data. The probability density functions (PDF) of partitioning distances with respect to each dataset differ significantly. The distribution for random training data is broader and more symmetric, whereas the text and image data have distributions that are more peaked and skewed, with the image data having a longer right-sided tail in its distribution, whereas the Wiki text distribution has a “shoulder” at small distances that does not go to zero, indicating clusters with significant overlap.

One of the goals of this paper is to test robustness and generalizability of the hierarchical linkage regression model with respect to various real-world data. Fig 5 depicts the L1 norm probability density functions of the partition distances for the texture image data, Wikipedia text data, and synthetic training data, respectively. As the plot demonstrates, the data points of the respective datasets come from substantially different distributions, providing assurance that the data selected for evaluation are adequate for purposes of testing generalizability of the regression model.

### 2.6 Comparison to established cluster number estimation methods

To compare performance of hierarchical linkage regression (HLR) to other established cluster number estimation techniques, test sets of *K* = 100 clustering instances of $\hat{y}=15$ randomly selected clusters built from (1) synthetic normally-distributed data, (2) texture image data and (3) Wikipedia embedded text data, were utilized. The high number of clusters was chosen to ensure the test would be sufficiently challenging to allow for greater discrimination between methodologies. The comparison was made for standard statistical and information-theoretic methods of estimating cluster number, and also for modern automated methods of clustering whereby cluster number is automatically estimated as part of the algorithm. In terms of practical implementation, the measures were computed using Python’s scikit-learn library and the Matlab computing environment (Natick, MA). The best performing models from this initial evaluation were then selected for more rigorous testing over a range of different cluster numbers (from 1 to 15) using 1000 randomly selected clustering instances in each case, or on average, over 60 different clustering instances per cluster number. The specific metrics used for evaluation are discussed below.

#### 2.6.1 Comparator methods

For statistical comparators, the silhouette method, the gap statistic, Davies-Bouldin criterion, and Calinski-Harabasz index were evaluated against hierarchical linkage regression [1–3,5]. The silhouette method is a measure of distance-based similarity of a given point to other within-cluster points versus points in external clusters, normalized to lie in the range from –1 to +1. The optimal cluster number is the one that produces the highest silhouette score averaged over all data points (i.e. maximizes separation between clusters and minimizes within-cluster separation). The gap statistic is a measure of the difference between the logarithmic within-cluster dispersion (pooled averaged distances of within-cluster points over all clusters in the dataset) and its expected value drawn from a reference distribution. The optimal cluster number corresponds with the smallest value, *ξ*, such that the gap statistic is within one standard error of the gap at *ξ* + 1. The Davies-Bouldin (DB) criterion is computed from ratio of within-cluster and inter-cluster distances whereby the optimal cluster number corresponds to the minimum DB value. Similarly, the Calinski-Harabasz index is computed as a ratio of inter-cluster variance to within-cluster variance, whereby good clustering solutions tend to have large inter-cluster variation and smaller within-cluster variation, so that the optimal cluster number corresponds to the largest ratio value obtained.

For modern automated clustering methods, HLR was compared to affinity propagation [7], DBSCAN [10] and OPTICS [9]. I will not go into significant detail about these methods, as those details are provided in the corresponding referenced literature. In brief, affinity propagation is a form of network clustering, whereby “messages” are sent between data points to determine exemplars representing the other data points and thereby defining clusters. The messages are divided between responsibility and availability for the *k*th point to be the exemplar of the *i*th point, and these are iteratively updated until convergence. A crucial parameter is the *preference*, which affects cluster number determination by controlling the number of exemplars allowed. The damping factor helps to smooth fluctuations in the iterative updating of availability and responsibility, helping convergence. DBSCAN and OPTICS are density-based clustering methods (clustering areas of high density in feature space, separated by areas of low density). DBSCAN uses a parameter, *epsilon*, to adjust the neighbourhood for finding other adjacent samples in determining density, and therefore the estimated number of clusters is quite sensitive to this parameter. OPTICS is a generalization of DBSCAN to whereby clusters are determined by analysing densities over a range of epsilon values (producing a “reachability” plot), with cluster boundaries being defined when the slope of the reachability curve surpasses a steepness threshold.

#### 2.6.2 Metrics for quantitative model evaluation

To quantify the performance of the cluster estimation methods, the recall (sensitivity) and Jaccard similarity index were calculated over each batch of *K* clustering instances for each model type and each dataset, over cluster deltas (Δ) from 0 to 5. Recall is defined by the ratio of true positives to the sum of true positives and false negatives:

$$recall(\Delta )=\frac{{TP}_{\Delta }}{{TP}_{\Delta }+{FN}_{\Delta }}$$ (18)

which in the binary case of all positive test cases (i.e. all clustering instances having ground truth cluster number equal to 15) is synonymous with accuracy. The Jaccard index is a measure of similarity between two sets and is defined by a ratio of the intersection to the union of the sets [30]:

$$J(A,B)=\frac{A\cap B}{A\cup B}$$ (19)

where for purposes of model evaluation the test set is a binary set corresponding to whether a given cluster estimate is within Δ of the ground truth (1) or not (0). The Jaccard index was normalized by the Jaccard index of the HLR model to obtain a relative model performance score:

$$\frac{J}{J_{HLR}}(\Delta )=\frac{(|Y-\hat{Y}|\leq \Delta )\cap 1}{(|Y-\hat{Y}|\leq \Delta )\cup 1}∙\frac{(|Y_{HLR}-\hat{Y}|\leq \Delta )\cup 1}{(|Y_{HLR}-\hat{Y}|\leq \Delta )\cap 1}$$ (20)

whereby a score equal to one implies parity in terms of model performance compared with the HLR model, a score greater than one, better performance, and a score less than one, worse performance.

The best performing models from the different categories of information-theoretic and automated clustering techniques were then tested further against HLR for the scenario incorporating multiple intrinsic cluster numbers $\hat{y}=\{1,2,\ldots ,{\hat{y}}_{max}\}$, by computing the cluster-number averaged F1 score for each cluster Δ from 0 to 5. The F1 score is the harmonic mean of the recall and precision, itself a derivation of the F-measure [31], with recall defined by Eq (18), and the precision defined by the ratio of the true positives to the sum of true positives and false positives,

$$precision(\Delta )=\frac{{TP}_{\Delta }}{{TP}_{\Delta }+{FP}_{\Delta }}$$ (21)

so that the binary F1 score takes the form,

$$F1(\Delta )=\frac{2∙precision(\Delta )∙recall(\Delta )}{precision(\Delta )+recall(\Delta )}.$$ (22)

For multiple intrinsic cluster numbers, the aggregate (macro) F1 score is then expressed as the mean of the cluster-number-specific F1 scores:

$$\overset{-}{F1}(\Delta )=\frac{1}{{\hat{y}}_{max}}\sum_{\hat{y}=1}^{{\hat{y}}_{max}}{\left[F1(\Delta )\right]}_{\hat{y}}$$ (23)

where ${\hat{y}}_{max}$ is the highest intrinsic cluster number in the test set. The F1 score ranges from 0 (worst) to 1 (best), penalizing both false positives and false negatives, and is a balanced measure of performance in circumstances where the number of negative cases greatly outnumbers positive cases, as occurs when associating positive cases with a specific cluster number within the multi-cluster number evaluation scenario. Similar to the F1 score, the normalized Jaccard index (Eq (20)) can be adapted for multi-cluster number evaluation by computing the mean of the binary Jaccard indexes over the range of cluster numbers tested, although this necessitates ground-truth labels incorporate zeros for negative cases instead of being entirely unity.

## Results

### 3.1 Inferring cluster number from linkage hierarchies

**Fig 6:**
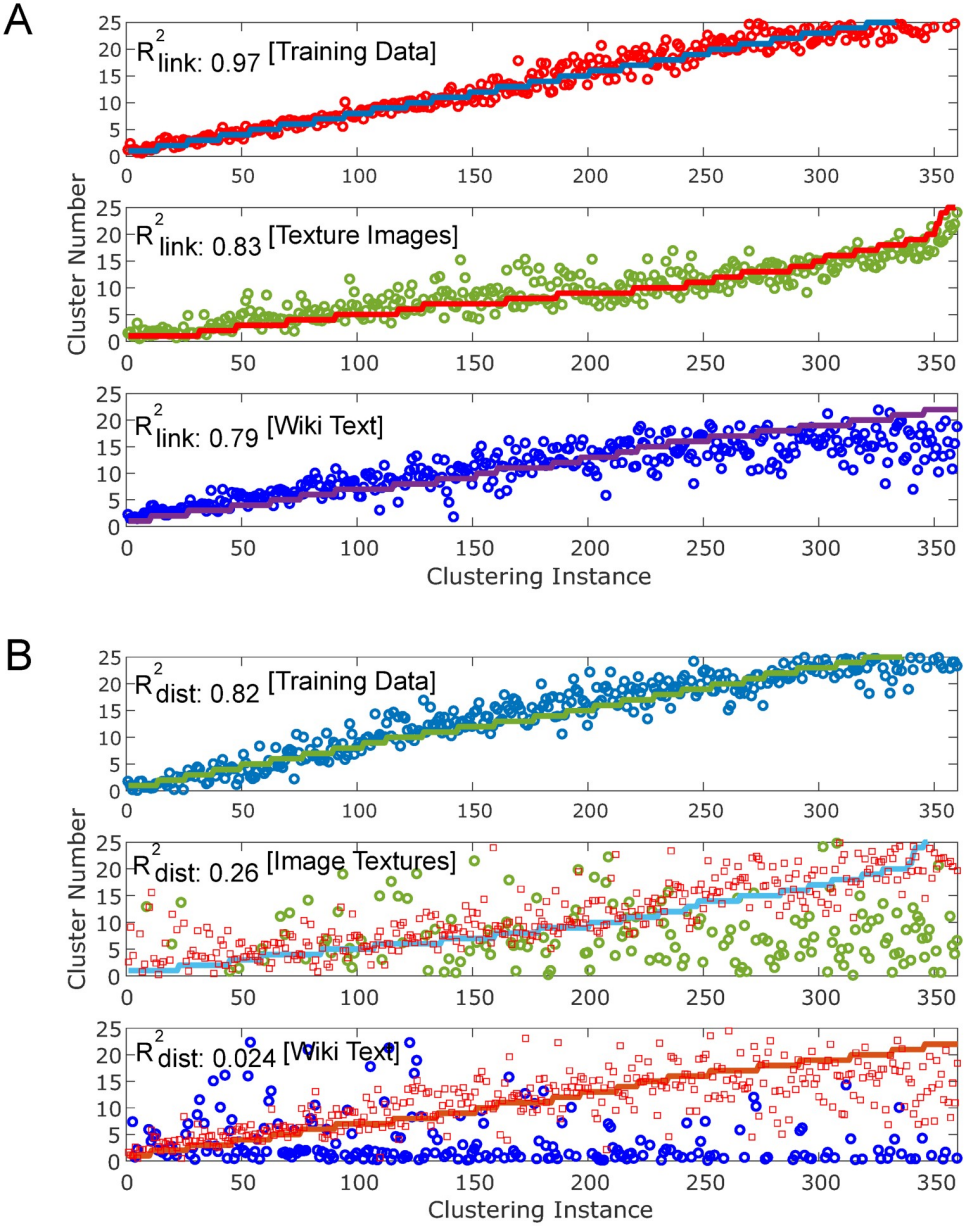
Estimation of cluster number by hierarchical linkage regression. (A) Results for cluster number estimation (inference) based on linkage hierarchy. Coefficients of determination (*r*^2^) are reported for each dataset. Top: fitted training set inferences (open circles) plotted against ground truth (solid stepped line); middle: image data inferences (open circles), plotted against ground truth (solid stepped line); bottom: Wiki text data inferences (open circles), plotted against ground truth (solid stepped line); note the underestimation of cluster number for text data at high cluster counts. Linkage type: complete; distance metric: L1 norm; histogram partitions: *R* = 40; embedding dimension: *d*_*E*_ = 50. (B) Results for cluster number estimation based on distance (L1 norm). Plot of training set (top) and test set (middle: image; bottom: text) cluster number inferences versus ground truth (solid stepped line), comparing distance-only (open circle) vs. linkage hierarchy (open red square) regression models, matched to have same input vector size and network architecture. The distance-only regression model fits the training data well, but is unable to generalize to image data or text data. Coefficients of determination (*r*^2^) are reported for the distance model only.

The regression model was trained on synthetic data using 3800 training samples, each consisting of between 1 and 30 random clusters of varying sizes and distribution, using the methodology described in the previous section. The number of bins used to create the 2-dimensional histogram of hierarchical linkage coordinates was 40 × 40 = 1600 (using *R* = 40; see Section 2.1) which was then reduced to 820 after removing zero values below the matrix diagonal. As seen in Fig 6A, the results of both the training and test sets show good correspondence in terms of the fit to cluster number. The regression plots were generated by re-ordering the samples according to increasing cluster number, with the corresponding model outputs overlaid against the reference “ground truth” line. Inferring cluster number was in general more robust at high cluster counts for the image texture data than for text data, especially when cluster number exceeded around 15 clusters or so. For text data in particular, the regression model underestimates the true cluster count above that level, whereas the model output for image data tracks ground truth closely even as cluster number exceeds 20 to 25 clusters. This is likely due to the somewhat arbitrary designation of text clusters by semantic similarity, which allows for significant cluster overlap and for smaller clusters to be embedded entirely within larger clusters of comparable sparsity. This effect can be seen in the probability density function for the text data in Fig 5, whereby there is a non-zero plateau at small distances compared with the other probability functions going to zero. The significant degree of cluster overlap due to subjectivity of the way text clusters were defined in terms of semantic similarity made cluster estimation for the text data very challenging, whereas the image clusters were defined in a non-arbitrary way by virtue of belonging to separate texture classes.

A regression model based on distance metrics rather than linkage hierarchies was trained to demonstrate that distance information alone is insufficient to infer intrinsic cluster properties. Training the FNN using a 200-bin histogram of the distances between cluster partitions, instead of the two-dimensional linkage coordinates, results in very poor performance. To make the comparison fair, a 210-bin histogram of linkage coordinates (*R* = 20) was used for the competing regression model, so that differences in feature vector length did not factor into differences in performance results. The model trained on the distances alone is able to fit the training set, but then completely fails to capture the relationship to cluster number when applied to the test datasets (Fig 6B). This is not the case for the model trained on linkage hierarchies. The failure of the distance model to generalize to real data supports the hypothesis that the two-dimensional linkage coordinates contain intrinsic information regarding organization of data into clusters not captured by the one-dimensional distance information.

**Fig 7:**
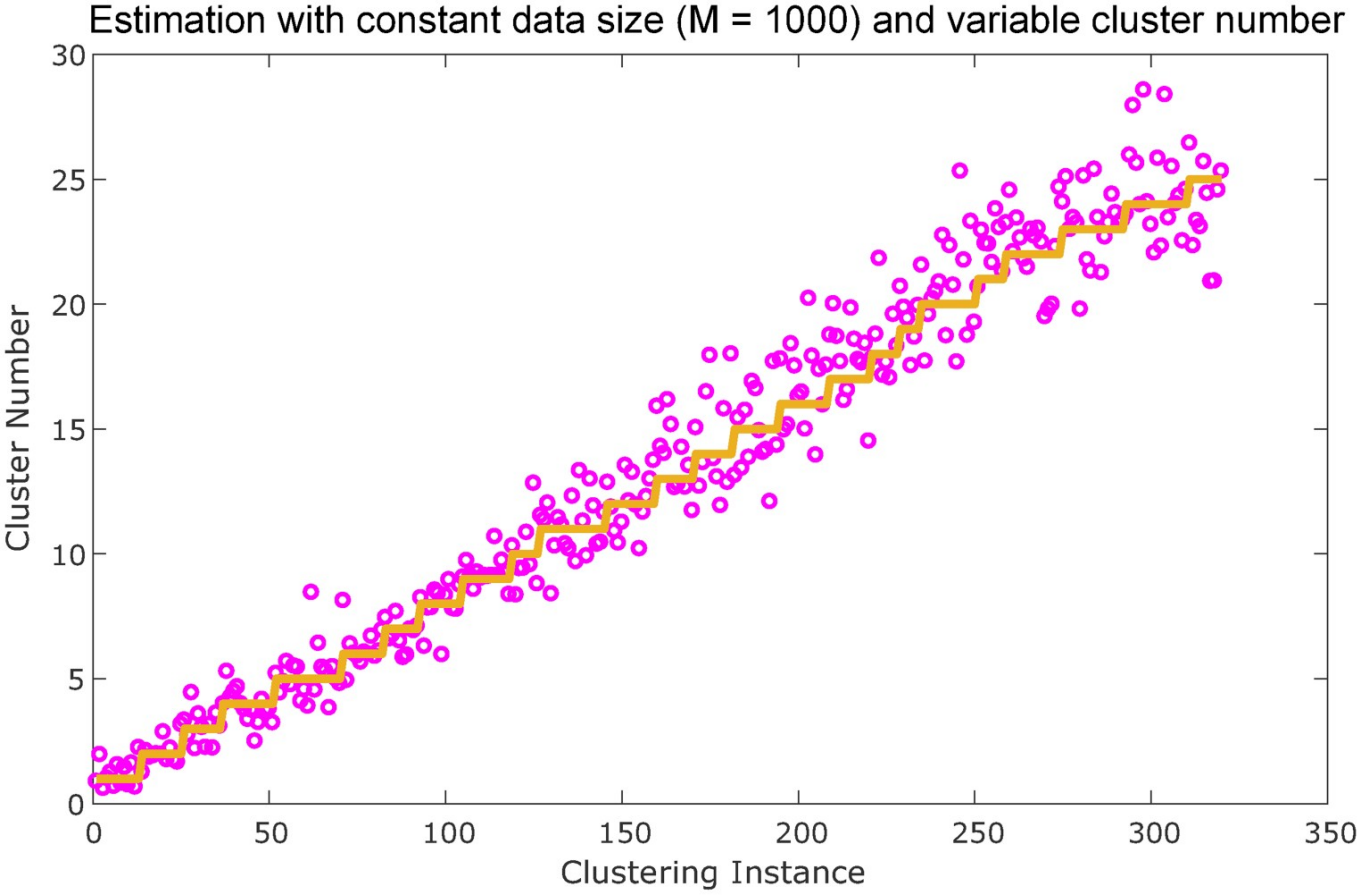
Cluster number estimation for constant number of data points. HLR was applied to a random set of clustering instances with variable cluster number (1 to 25), but the total number of data points was fixed at *M* = 1000 for all clustering instances. The plot shows the estimates of cluster number vary 1:1 with the ground-truth values, confirming HLR does not infer cluster count based on number of data points present, which is a possible confounder because the total number of data points would be expected to scale proportionally with the number of clusters present.

To ensure the model is inferring cluster number by linkage hierarchy, and not merely the number of data points present (because a larger number of clusters would be expected to have more data points), a constant sized test set was generated (Fig 7), whereby the number of clusters was allowed to vary over the range of 1 to 25, but the number of data points was kept constant at 1000. In this way, the model had to infer the correct number of clusters whether hypothetically for a sample case of 2 clusters comprising 376 and 624 data points, respectively, or a set of 14 unequal clusters whose total collection of points sum to 1000. As Fig 7 shows, the regression model performs expectedly, supporting the hypothesis that cluster number inference is based on linkage hierarchy and not the absolute number of data points present.

### 3.2 Comparison to other cluster number estimation methods

#### 3.2.1 Single cluster number evaluation

Performance of comparator methods was tested against HLR on *K* = 100 clustering instances, with ground truth of $\hat{y}=15$ clusters across all instances, as described in Section 2.6. For statistical methods requiring specification of cluster number as an input parameter, trial clustering of the data was iteratively performed over cluster numbers ranging from *k* = 1 to 30, and the estimate associated with the optimal clustering solution selected. These models were tested using both k-means and hierarchical clustering because some methods are better suited to use with k-means, such as the Calinski-Harabasz index. For the modern automated clustering methods, parameters were pre-tuned on the normally-distributed synthetic random data, because those data are considered “ideal”, whereas model parameters were not re-tuned when applied to the texture data and Wikipedia text data, for which *a priori* knowledge of clustering properties of the data was assumed to be unavailable.

From the pre-tuning results, both affinity propagation and DBSCAN were found to be highly dependent on selection of the *preference* and *epsilon* parameters, respectively. According to literature, a reasonable initial value for *preference* for affinity propagation (AP) is the negative of the squared Euclidean distance (ED^2^) of the points farthest away from each other in the set (the negative squared L2 norm of the distances serves as a similarity matrix used by the algorithm). The AP algorithm tended to overestimate cluster number so a range of *preference* values was sampled ranging from the negative of the median[ED^2^] to negative 5×max[ED^2^], picking the minimum cluster number estimate obtained. The damping factor was set to 0.9, which gave significantly more stable and robust estimates than the default (0.5). For the DBSCAN method, the *epsilon* value was set to be equal to a fraction of the maximum Euclidean distance, in this case 0.384× ED, setting 10 to be the minimum number of sample points to form a cluster, which gave the correct cluster number estimate of 15 for the ideal clustered data. OPTICS, as well as most standard cluster number estimation techniques, gave reasonable estimates for the ideal clustered data without any tuning, and therefore default parameters were used.

**Fig 8:**
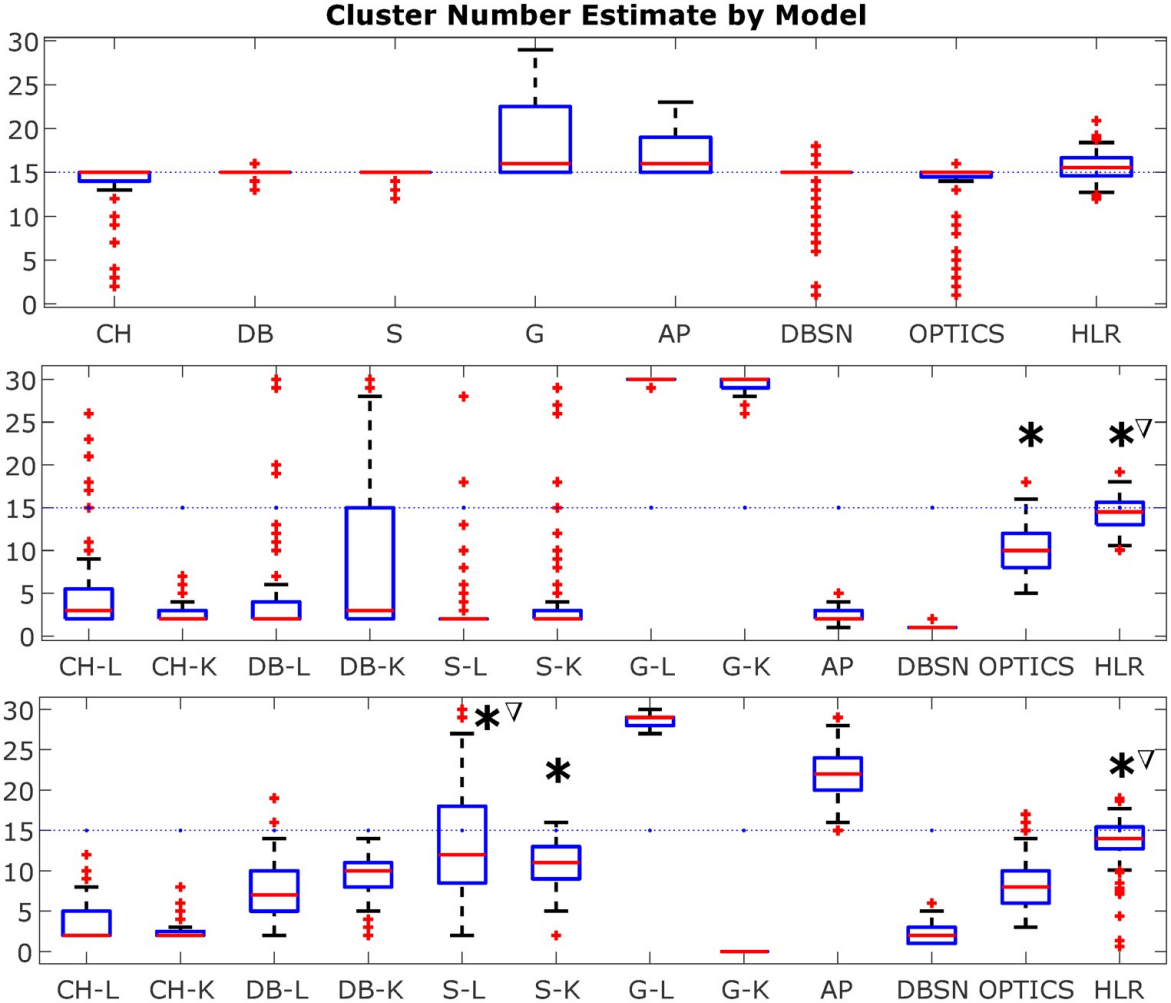
Boxplot comparing cluster number estimation models for single cluster number scenario ($\hat{y}=15$). The upper, mid and lower plots pertain to results for ideal reference data, texture image data, and Wikipedia embedded text data, respectively, representing 100 clustering instances in each case. Ground truth is $\hat{y}=15$ clusters (blue dotted line). Asterisks for image and text data boxplots denote statistical significance with respect to the distribution of HLR being different from comparator models without asterisks, by the Friedman test (*p* ≪ 0.0001). Triangles denote statistical significance by one-way ANOVA for comparison. Boxes: lower bound Q1 quartile, upper bound Q3 quartile; whiskers: length bounded by (Q3-Q1) interquartile range. Model abbreviations: Calinski-Harabasz (CH); Davies-Bouldin (DB); Silhouette (S); Gap (G); Affinity Propagation (AP); DBSCAN (DBSN); OPTICS (same); HLR (hierarchical linkage regression); linkage clustering (-L); k-means clustering (-K).

Fig 8 is a boxplot of the model runs for ideal data (normally-distributed random data), texture image data, and Wikipedia embedded text data, respectively, with ground truth being equal to 15 clusters in all instances. Results are also summarized in S1 Table in the Supporting Information section. For the ideal data, there was little variation between models, with the majority of models giving accurate estimates of cluster number, although some models had greater dispersion in their estimates, including the gap statistic and affinity propagation, with tendency to overestimate the value. Furthermore, several of the statistical methods (silhouette, Davies-Bouldin and Calinski-Harabasz) and automated methods (DBSCAN and OPTICS) had tighter deviations on the estimate of cluster number than hierarchical linkage regression. However, when comparing model performance on image texture data and Wikipedia text data, HLR was superior to other models by a significant margin, being the only model to give reliable estimates of the true cluster count, whereas other models struggled to produce accurate estimates, mostly underestimating the cluster number for both the image and text data. A couple of models performed more unpredictably, with affinity propagation underestimating the number for texture data, but then overestimating it for text data, and similarly for the gap statistic using k-means (G-K). Only the silhouette method using hierarchical linkage clustering (S-L) produced estimates in the vicinity of the ground truth for Wikipedia text data, although there was a wide dispersion on the estimates.

**Fig 9:**
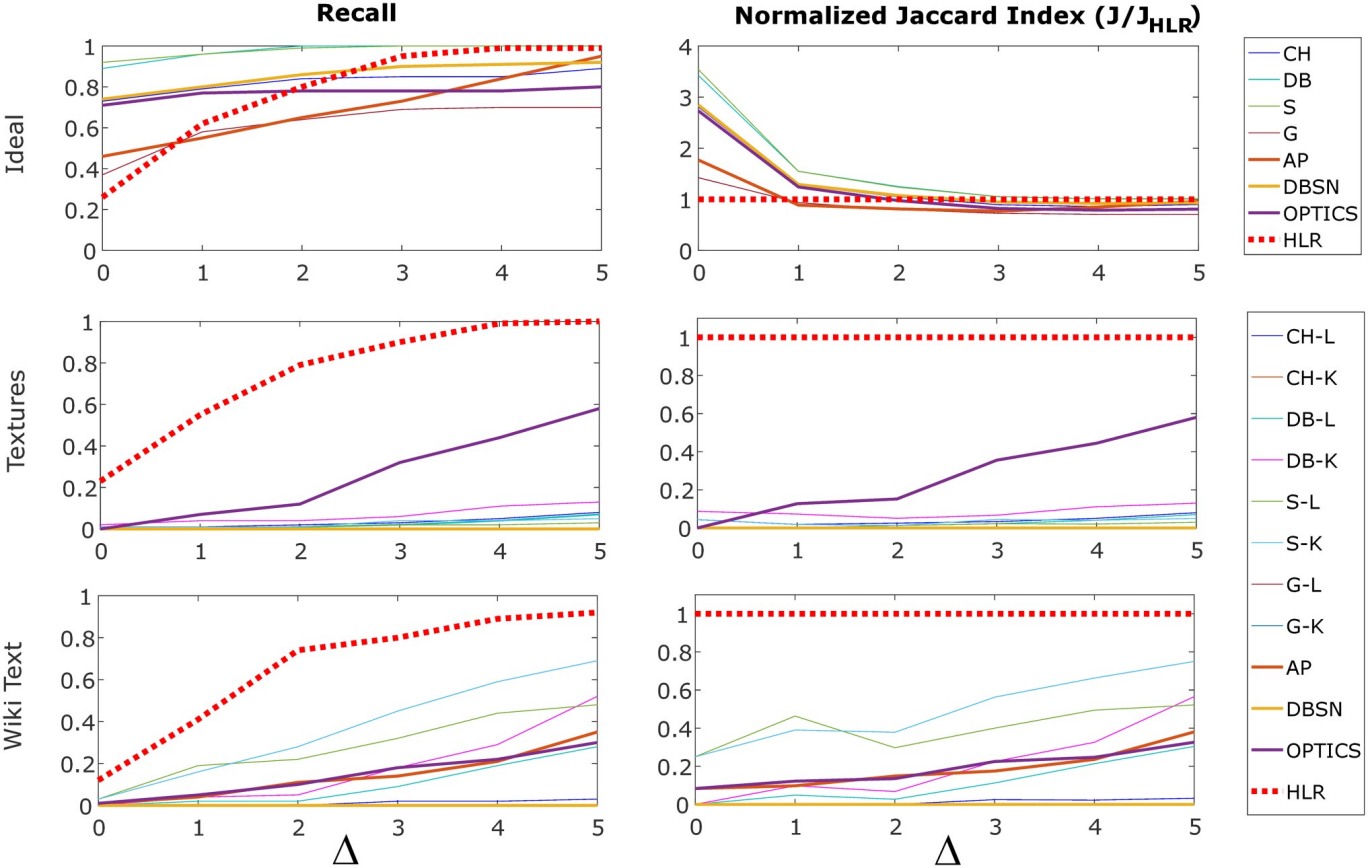
Quantifying performance (recall and normalized Jaccard index) for single cluster number scenario ($\hat{y}=15$). The upper, mid and lower plots pertain to results for ideal reference data, texture image data, and Wikipedia embedded text data, respectively. Values of recall and normalized Jaccard index are plotted with respect to the estimate being within Δ of the ground truth number of clusters ($\hat{y}=15$). Model abbreviations: Calinski-Harabasz (CH); Davies-Bouldin (DB); Silhouette (S); Gap (G); Affinity Propagation (AP); DBSCAN (DBSN); OPTICS (same); HLR (hierarchical linkage regression); linkage clustering (-L); k-means clustering (-K).

Further quantification using *recall* and the *normalized Jaccard similarity* measures (Fig 9) again show comparators mostly performed on par with the HLR model for ideally clustered data, although there is some variation with cluster delta (Δ), in that HLR performed worse than other models for small Δ, and was on par with or superior to other models for Δ > 1. However, with respect to the image and text data, both measures demonstrate consistency and superiority of the HLR model in generating accurate cluster number estimates, in particular for Δ > 1, with HLR model performance being superior to other models over the entire range of Δ. Results for recall are tabulated in S2–S4 Tables, and differ significantly across models by Friedman’s test, a ranked non-parametric alternative to ANOVA valid for repeated measures [32] (ideal data: *χ*^2^-statistic 34.7, *p* = 1.3 × 10^−5^; image data: *χ*^2^-statistic 59.4, *p* = 1.2 × 10^−8^; Wiki text: *χ*^2^-statistic 62.2, *p* = 3.6 × 10^−9^).

#### 3.2.2 Multiple cluster number evaluation

The best performing comparator models in the single cluster number evaluation scenario were silhouette and Davies-Bouldin in the statistical/information-theoretic category, whereas OPTICS was the best performing automated clustering method. Therefore, these models were selected for more rigorous multi-cluster number testing, as described in Section 2.6. As such, *K* = 1000 clustering instances were generated each for normally-distributed random (ideal) data, texture data and Wiki text data, respectively, with over 60 instances per intrinsic cluster number, $\hat{y}$, ranging over 1 to 15 clusters.

**Fig 10:**
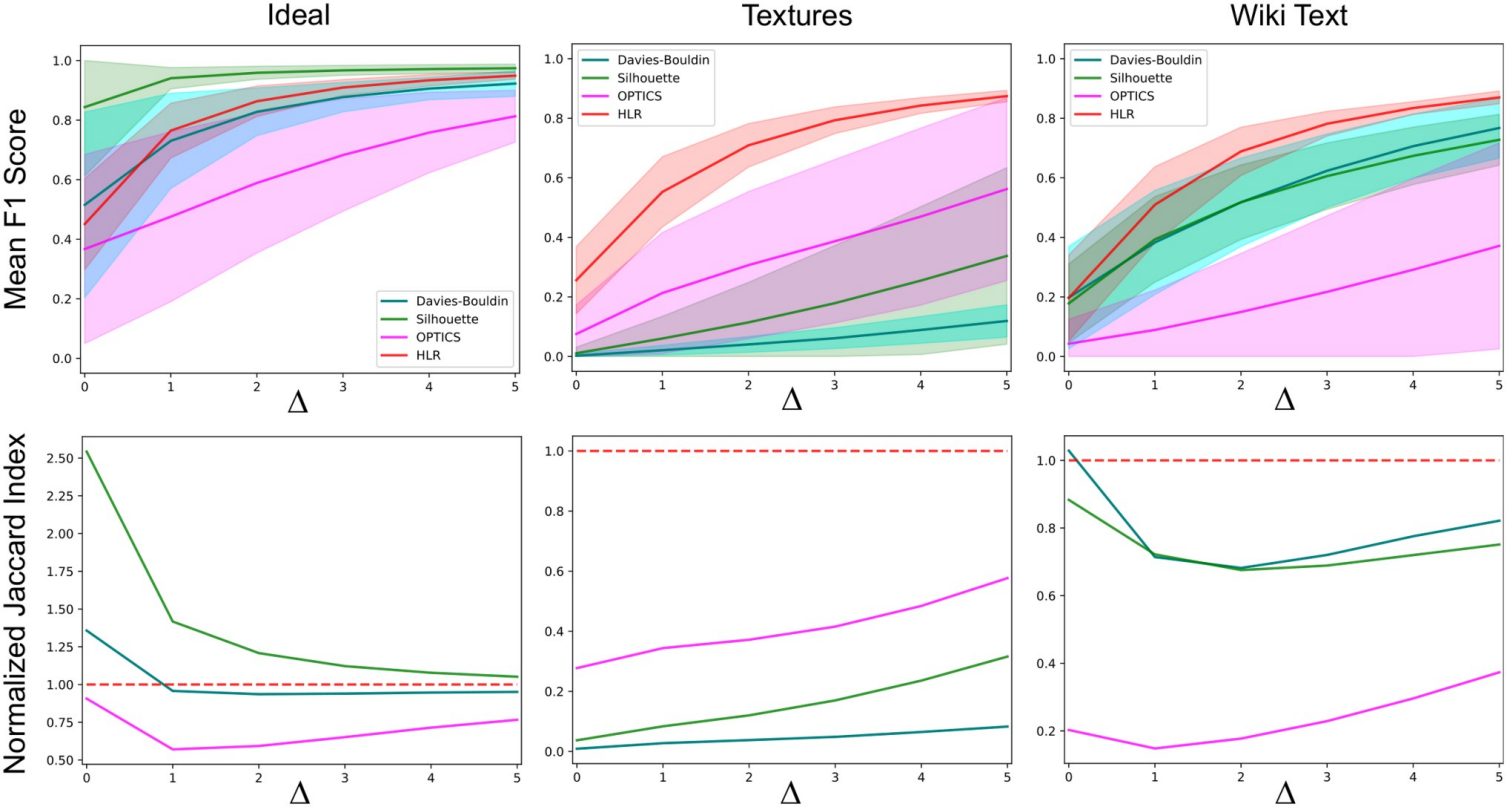
Quantifying performance (mean F1 score and normalized Jaccard index) for multi-cluster number scenario ($\hat{y}$ ranging from 1 to 15). Top row: mean F1 score of cluster number estimate being within Δ clusters of the ground-truth, for ideal, image and text data, respectively, testing the best-performing comparator models to hierarchical linkage regression (Davies-Bouldin, Silhouette and OPTICS), as determined from single cluster performance evaluation. Colour shaded regions represent standard deviation of the mean F1 score. Bottom row: mean normalized Jaccard index, corresponding to the same models tested in the multi-cluster number evaluation. The results for the comparators are normalized against the Jaccard index for HLR so that the reference is unity (segmented red line). The results of the F1 score and normalized Jaccard index reveal HLR outperforms its comparators on real-world text and image data, and has lower variance in performance measures, implying greater consistency in its performance across different cluster numbers and cluster deltas.

Results of the mean F1 score and normalized Jaccard index for the multi-cluster number evaluation are depicted in Fig 10. They show that HLR is robust and consistent in terms of its performance across datasets. As well, the standard deviation of F1 scores for HLR is narrow compared to other models, especially OPTICS, which had wide variation in F1 score due to significantly better performance for smaller cluster number than in instances where cluster number was larger than 10 or so. For the ideal data, HLR was not the best performing method, but was comparable to the other methods. For texture image data, the closest to HLR in terms of performance was OPTICS, but the method still performed significantly worse than HLR, whereas for Wiki text data, the statistical methods (silhouette and Davies-Bouldin) were comparable to each other and superior to OPTICS, but inferior to HLR. F1 scores were significantly different across the models by Friedman’s test (ideal data: *χ*^2^-statistic 198.1, *p* = 1.1 × 10^−42^; image data: *χ*^2^-statistic 193.1, *p* = 1.3 × 10^−41^; Wiki text: *χ*^2^-statistic 192.9, *p* = 1.5 × 10^−41^). In general, the results of multi-cluster number evaluation did not differ significantly from the results of single cluster number evaluation, with HLR being the most consistent and robust performing method overall across the datasets tested.

#### 3.2.3 Comparative performance on other real-world datasets

HLR was tested on the benchmark iris, wine, glass, and spam datasets obtained from the UCI machine learning repository [20]. The results are presented in Table 1. To assess variability owing to model initialization and training, one hundred regressors were trained on the same synthetic training dataset in each case, but with different random initializations of model coefficients. As well, a stochastic solver for backpropagation (Adam [33]) was used to train each FNN, which had the same architecture as in Fig 2A, but with rectified linear (ReLu) hidden-layer activation functions. An embedding dimension of *d*_*E*_ = 100 was used for the spam dataset, which has feature dimensionality of 57, since the embedding dimension of 50 used for the other datasets was not sufficient to accommodate the higher-dimensional dataset. In terms of performance, HLR was able to accurately infer the number of classes in each case to within a rounding error. The ground truth value could be retrieved by taking the median of the rounded inference values, since raw outputs are continuous. Comparison of HLR to other methods was not explicitly carried out in this paper, because detailed analysis of comparators on these datasets using randomized initialization was already performed by Flexa et al. [6], to which I refer the reader.

**Table 1:** Cluster number inference by hierarchical linkage regression for other real-world data (data source: UCI machine learning repository [20]).

Dataset	Number of datapoints	Data dim	Embedding dim	Model raw inference	Median rounded	Ground truth
**wine**	59+71+48 = 178	13	50	2.63 ± 0.31	3	3
**glass**	70+17+76+13+9+29 = 214	9	50	6.40 ± 0.59	6	6
**iris**	50+50+50 = 150	4	50	2.66 ± 0.49	3	3
**spam**	1813+2788 = 4601	57	100	2.06 ± 0.14	2	2

*N* = 100 randomly initialized regressors. *Model raw inference* reported as mean ± std dev. *Median rounded* refers to median of the rounded inference values (to nearest integer).

### 3.3 Sensitivity analysis

Dependence of HLR model performance on its parameters was evaluated by examining the percentage of samples lying within Δ clusters of ground truth, over the range of Δ = 0 to 5, plotted for different parameter values. Regression uncertainty was quantified through estimation of confidence intervals. A percentile bootstrap method was used to generate 200 test cases of 200 samples each with replacement in order to estimate the confidence bounds on regression [34].

#### 3.3.1 Effect of cluster number on performance

**Fig 11:**
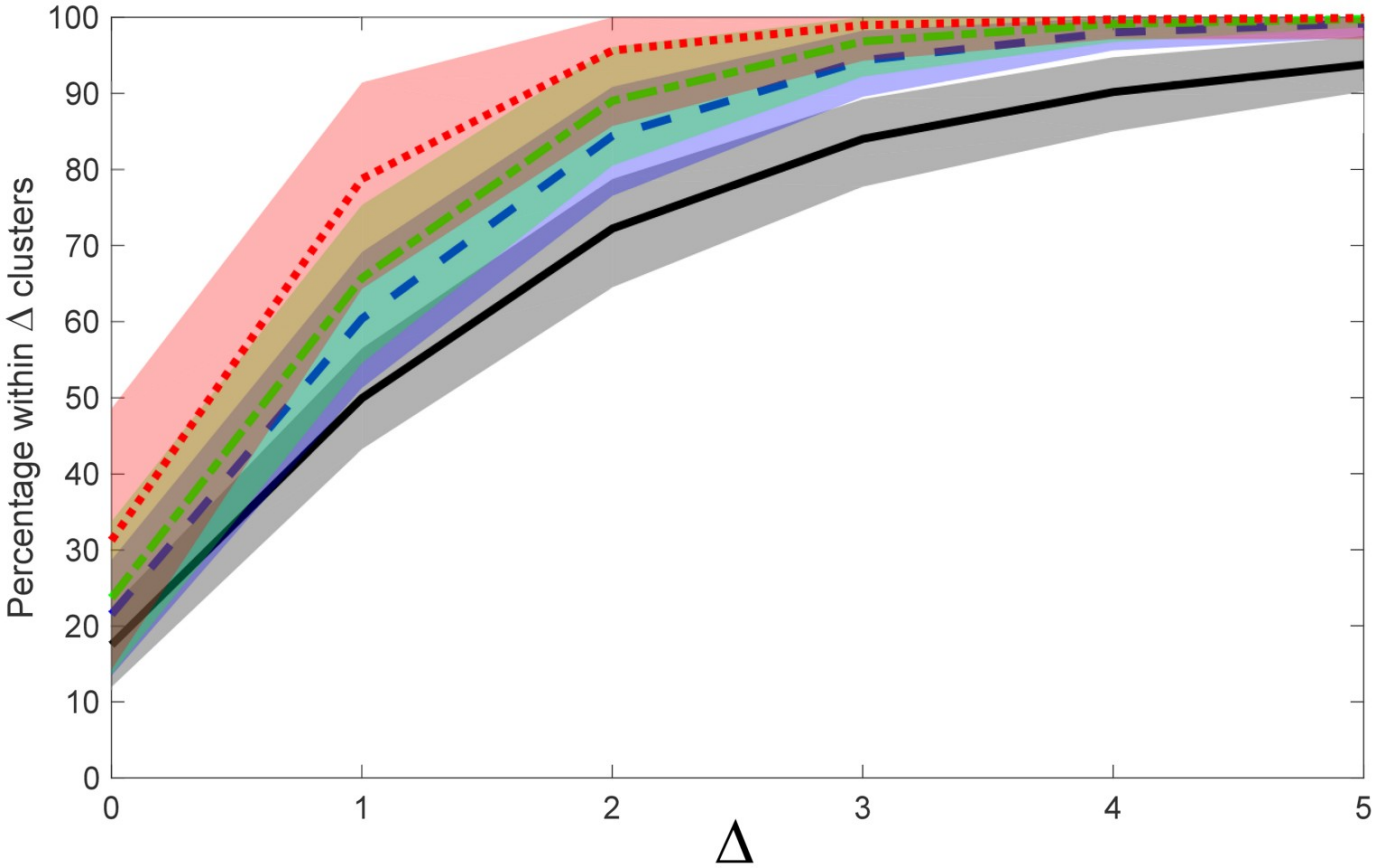
Effect of cluster number on model performance (Wikipedia data). Colour shaded regions represent 95% confidence interval with respect to mean percentage of model estimates lying within clusters of the true cluster number (corresponding coloured line), for text data only. Legend: dotted RED–estimates for samples containing up to 5 clusters; short-long segmented GREEN–estimates for up to 10 clusters; segmented BLUE–estimates for up to 15 clusters; solid BLACK–estimates for up to 20 clusters. Distance metric: L1 norm; histogram partitions: R = 40; embedding dimension: d_E_ = 50.

As seen in Fig 6A, the model performance itself can exhibit dependence on cluster number. Although not prevalent for normally-distributed reference data or image texture data, the model exhibited a drop in performance for Wiki text data when cluster number exceeded 15 or so. Fig 11 quantifies this dependence by plotting percentage curves of clusters lying within Δ of ground truth and their associated confidence intervals, for test cases containing up to a maximum of five, ten, fifteen and twenty clusters, respectively. The results confirm a significant drop in performance for maximal cluster counts greater than 15 for Wiki text, reflecting an underestimation of the true number of word clusters present. As explained previously in Section 2.5, this is likely partially due to the arbitrariness of defining word clusters by semantic similarity, which allows for significant degree of cluster overlap, thereby impacting upon cluster estimation accuracy when cluster number (or cluster density) is high. When cluster number is approximately 15 or lower, the performance results for Wiki text approach those of the image and reference data.

#### 3.3.2 Effect of linkage type and distance metric

**Fig 12:**
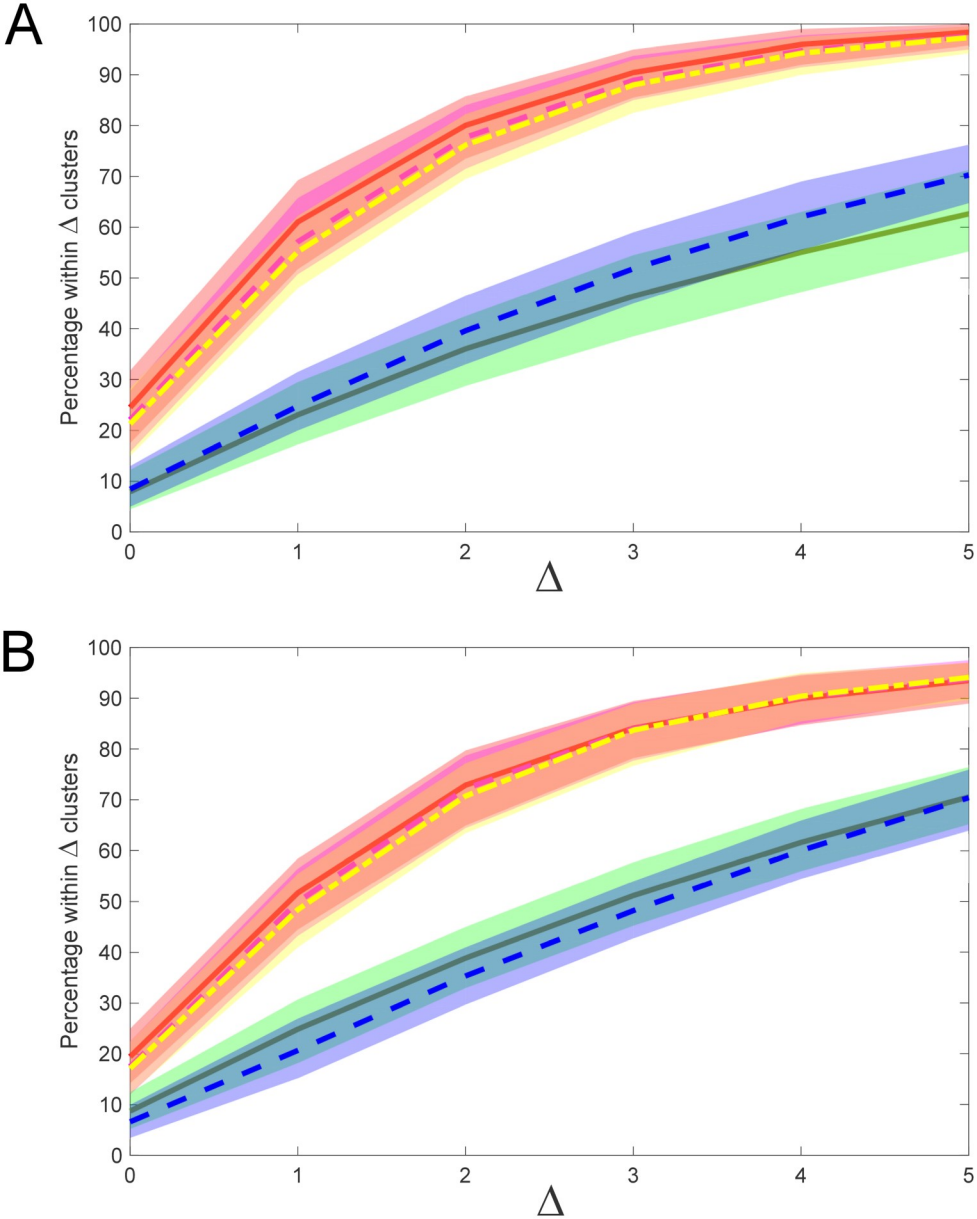
Effect of linkage type and distance metric on model performance. (A) Image data results; (B) Text data results. Colour shaded regions represent 95% confidence interval with respect to mean percentage of model estimates lying within Δ clusters of the true cluster number (corresponding coloured line). Legend: solid RED–complete linkage, L2 (Euclidean) norm; segmented MAGENTA–complete linkage, L1 (Manhattan) norm; short-long segmented YELLOW–Ward’s method; solid GREEN–single linkage, L2 norm; segmented BLUE–single linkage, L1 norm.

Fig 12 demonstrates the effect of different linkage types (single, complete and Ward’s linkage) on regression performance for both L1 norm (Manhattan) and L2 norm (Euclidean) distance metrics. The best performing model was the complete linkage with L2 norm, however there was no major difference between complete linkage and Ward’s linkage in terms of performance, nor between L1 or L2 norms, with considerable overlap of confidence bounds for the 3 models (Ward’s method only uses the L2 norm by definition). The single linkage models performed significantly worse, and there was no significant difference between L1 and L2 norms, although single linkage with L1 norm performed slightly better on image data and slightly worse than single linkage with L2 norm on text data. Although single linkage is the simplest both computationally and conceptually, its simplicity does not offer sufficient power to accurately resolve clustering patterns.

#### 3.3.3 Effect of histogram partition size

**Fig 13:**
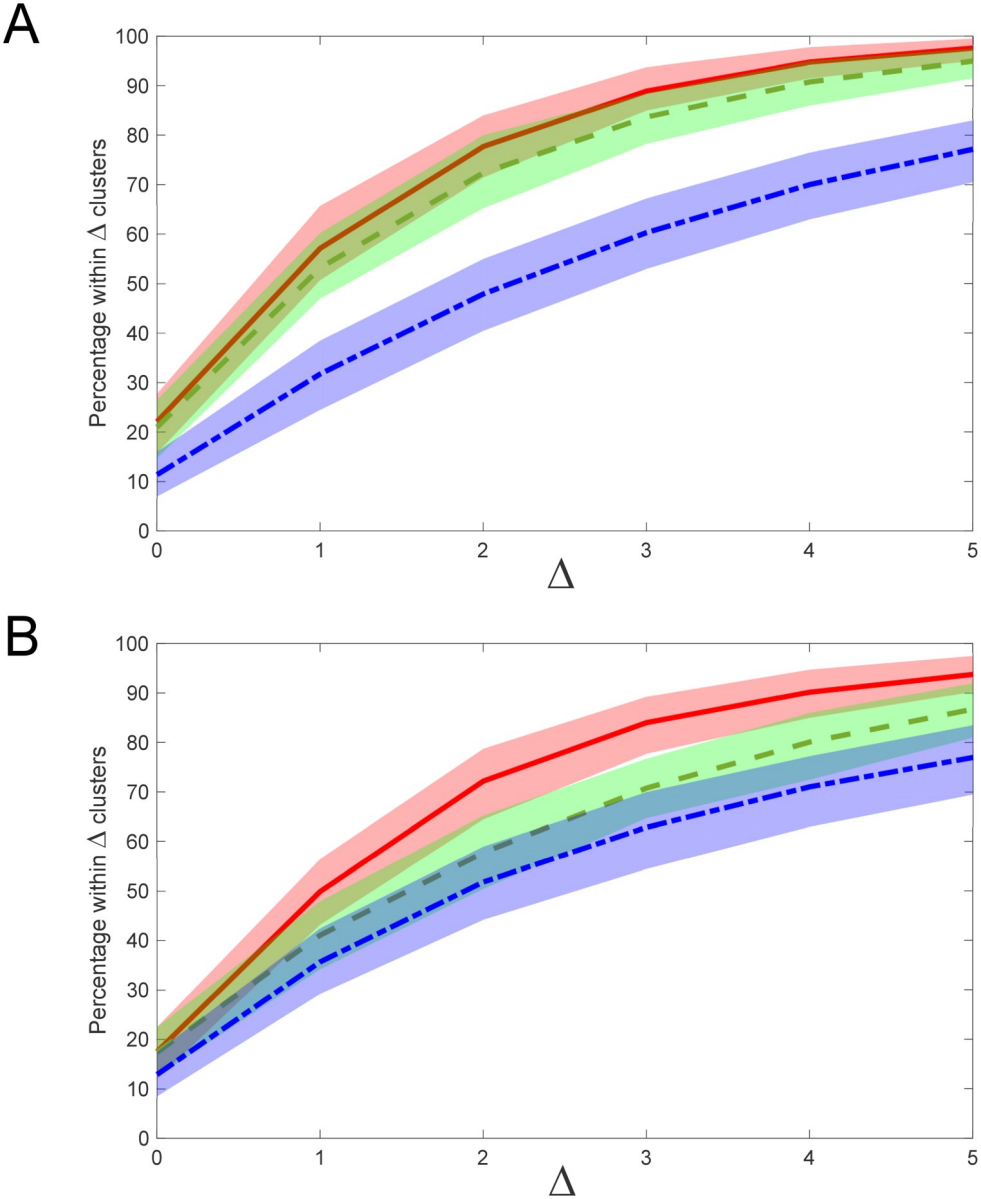
Effect of histogram partitioning (*R*) on model performance. (A) Image data results; (B) Text data results. Colour shaded regions represent 95% confidence interval with respect to mean percentage of model estimates lying within Δ clusters of the true cluster number (corresponding coloured line). Legend: solid RED, *R* = 40; segmented GREEN, *R* = 20; short-long segmented BLUE, *R* = 10. Distance metric: L1 norm; embedding dimension *d*_*E*_ = 50.

The parameter, *R*, defined in Section 2.1, governs the number of bins partitioning the linkage histogram, which in turn determines the granularity of the histogram’s two-dimensional distribution. As Fig 13 demonstrates, performance improves as bin number increases. The effect was more graded for the text data (Fig 13B), likely due to better resolution of embedded or overlapping clusters prevalent in the text data with increasing bin number. This intuitively makes sense as local variations in the linkage coordinates are better captured by the statistics computed on a finer partitioning grid.

#### 3.3.4 Effect of embedding dimension

**Fig 14:**
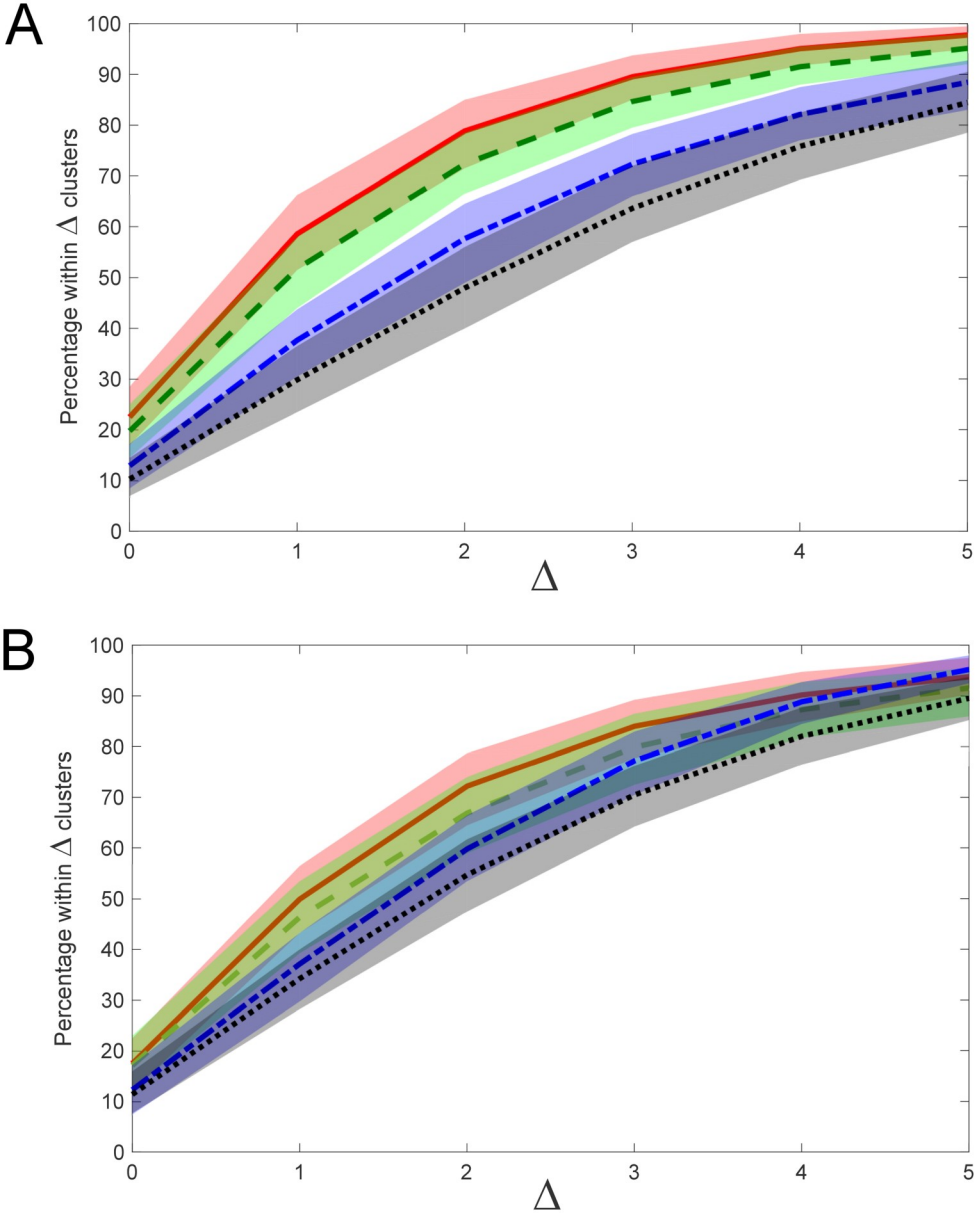
Effect of embedding dimension (*d*_*E*_) on model performance. (A) Image data results; (B) Text data results. Colour shaded regions represent 95% confidence interval with respect to mean percentage of model estimates lying within Δ clusters of the true cluster number (corresponding coloured line). Legend: solid RED, *d*_*E*_ = 50; segmented GREEN, *d*_*E*_ = 40 (image data); *d*_*E*_ = 30 (text data); short-long segmented BLUE, *d*_*E*_ = 10; dotted BLACK, *d*_*E*_ = 2. Distance metric: L1 norm; histogram partitioning: *R* = 40. Dimensionality of image data: 40; dimensionality of text data: 25.

It is important to select an appropriate embedding dimension (*d*_*E*_) for the training data so that the regression model is able to span the dimensionality of the experimental data. Results of sensitivity analysis indicate that embedding dimension of the training set should be at minimum equal to the dimensionality of the experimental dataset, or larger. Using a training set dimension significantly smaller than the dimensionality of the data being analysed yields poorer model performance in terms of estimating cluster number. On the other hand, using embedding dimensions slightly larger than the dimension of the experimental data does not appear to hurt performance, and may improve it. Fig 14A shows the correspondence of embedding dimension with improved model performance for embedding dimension meeting or exceeding dimensionality of the test data, which in this case are image data of dimension *n* = 40, while Fig 14B shows a similar result for embedded text data of dimension *n* = 25.

## Discussion

The key result of this study is that the hierarchical organization of data into partitions based on their relative separations in feature space is informative of the true clustering patterns within the data, and enables intrinsic properties of the data–such as number of clusters present–to be inferred by regression. The actual distances between data points in the feature space are only required to build the hierarchy, which is represented structurally by a binary tree (a dendrogram), and are not required to perform the regression. In fact, generating the histogram directly from the distance data to do the regression leads to poor results (Fig 6B), suggesting that it is the relative hierarchy of the data points and not their specific values or separations that is most important to enable inference of cluster properties. When tested on unrelated texture image datasets and Wikipedia text dataset, hierarchical linkage regression significantly outperforms other contemporary cluster number estimation methods, including statistical methods and network or density-based automated clustering methods, without the need to retrain the model or modify parameters (Figs 8–10). The model is also able to make appropriate inferences about cluster number in other real-world datasets (Table 1). The fact that the HLR model is able to generalize from the synthetic training data to experimental data of different data types, with distributions that vary considerably from one another (Fig 5), suggests that the relationship of linkage hierarchy to the intrinsic organization of data might have inherently conserved features that can be reliably exploited for the purposes of data mining and discovery.

The problem of determining intrinsic number of clusters in a multidimensional dataset is non-trivial. The method proposed in this paper uses regression, which has been shown to be a robust method for inferring cluster number, and enables quantification of uncertainty bounds on the estimate, given the assumption that the training and test data are distributed similarly (and the method has been shown to work even if the distributions do differ). Nevertheless, the uncertainty in the estimate implies the method may not yield an optimal solution in any one specific instance. Strategies to deal with this include hyperparameter tuning of the regression model and cross-validation on various benchmark datasets. If the measure is applied repeatedly through the training of multiple regressors, or by re-sampling the data, or by applying the measure to many similar datasets, then a more reliable “global” estimate may be obtained.

With an ability to determine uncertainty bounds on the estimate, the method can be seen as narrowing the search space that would otherwise need to be explored in clustering an unknown dataset. This becomes important as data dimensionality and number of clusters increases, given that difficulty and computational complexity often scale non-linearly with these factors. *A priori* knowledge is not needed about the variation or distribution of individual clusters within the experimental dataset. The algorithm is capable of estimating intrinsic cluster properties from the aggregate distribution by linkage analysis.

Another strength of the regression method is that it does not need to be trained on empirical data, because it can be deployed after training entirely on synthetic data. In this way, there is no limitation on data availability for training purposes, which is often a problem affecting other machine learning applications, leading to overfitting and poor generalizability. The method can be applied “blindly” to new data with expectation of reasonable results. Furthermore, the method appears robust to heterogeneity in the data, as demonstrated by the good performance of the model despite test data drawn from non-normally distributed text and image sources containing overlapping clusters, outliers and noise. Part of the reason for this robustness is that noise is inherently part of the training data, which are generated from random distributions, so the regression can readily accommodate randomness encountered in the experimental data. This is in contrast to other statistical or information-theoretic approaches, whose validity can diminish in the presence of noise or outliers within a dataset [35].

In terms of limitations, the proposed method is mainly limited by its computational complexity. Generating linkage hierarchies can be computationally expensive, the asymptotic time complexity being *O*(*m*^2^ log *m*), so this can become problematic for very large datasets. Training a regression model also can be computationally expensive, and in the case of neural networks, time complexity of backpropagation is *O*(*m* ⋅ *n* ⋅ ∏_*i*_ *h*_*i*_ ⋅ *e*), where *m* is the number of data points, *n* is the feature dimensionality, *h*_*i*_ is the number of hidden neurons in the *i*th hidden layer, and *e* is the number of epochs. Fortunately, there have been developments in terms of faster algorithms for hierarchical clustering [36], as well as other methods for speeding up the computation such as parallelization and GPU computation [37]. However, scalability of the method was not investigated in this study, as doing so would require modifications to implementation including hardware (e.g. GPUs) and larger datasets, and was not an objective of this proof-of-principle study. In principle, however, the method should scale to large datasets with appropriate modifications, because the algorithms underlying the method, namely linkage analysis and neural network regression, both independently scale well to large datasets. Nonetheless, if one were to consider using this method for much larger datasets without modification, then a possible approach might be to apply resampling methods to the entire dataset, to get a distribution of cluster estimates that might enable determination of a robust population estimate, but one would have to be cognizant of the limitations and biases of the resampling method chosen. The drawback is that such an approach may not be computationally efficient.

Another limitation of the hierarchical linkage regression model is the need to pick appropriate regression parameters, some of which are dependent on the specifics of the experimental data being modelled. Although the properties of the experimental data need not be fully characterized in order generate an adequate regression model, parameter selection concerning linkage type, embedding dimension, and histogram partitioning nonetheless impact performance (Figs 12–14). Fortunately, as the results of sensitivity analysis demonstrate, performance tends to be robust over a wide parameter selection range. Nevertheless, as a rule of thumb, one should pick an embedding dimension equal to or slightly larger than the dimensionality of the experimental data, and should also strive to create a synthetic training dataset with number of data points at least on par with number of data points in the experimental dataset.

Another factor possibly limiting model performance is the lack of hyperparameter tuning. Specifically, the neural network hyperparameters were not tuned for optimal model performance, given the purpose of the study was hypothesis testing and concept validation, and not practical application. It is quite possible that improved performance could be brought about by experimenting with different neural network architectures (e.g. number of hidden layers, number of neurons in a given layer, activation function type, etc.), or even with different regression models altogether, although this is beyond the scope of this study. One potential benefit of using a different regression model, such as a support vector machine instead of a neural network for example, is that the coefficients of regression might be more readily accessible. This in turn might make it easier to quantify regression uncertainty, rather than the bootstrapping method used here in this study, which comes with its own pitfalls and biases [38]. However, the trade-off in making a simpler model might be the negation of any performance benefit, or even a worsening of performance, because of poor model generalizability or decreased capacity to account for nonlinearities inherent in the data.

The cost function utilized in the backpropagation for training the regression model is also an area where efforts on improvement can be focussed. The current implementation uses a mean squared error (MSE) function with regularization, which is a classic loss term for regression problems. However, the MSE function treats the contribution from each output equally in terms of minimizing the quadratic error, and is prone to bias from outliers in the training data, which could affect performance. Better performance might be achieved by utilizing more tailored cost functions, for example, by weighting the contributions from regions of the curve that are more challenging to fit (such as the tail at higher cluster numbers), or using other cost functions like the mean absolute error or maximum error, although the latter have their own issues with respect to gradient computation for purposes of backpropagation and/or cost minimization.

Aside from model architecture and cost function selection, there are other more specialized ways the model might be improved in future implementations. For instance, the experimental distributions for test data in this study differ from the distribution of the training data. On one hand, this can be considered a strength of the method, since it demonstrates the ability of the model to generalize to dissimilar data. However, the result also suggests a possible means of improving performance using an inverse approach: namely, by first measuring and fitting the statistical distribution of the experimental data, and then using the fitted distribution to synthesize the training set for regression, instead of the current method of synthesizing the data from an arbitrary random distribution, and then applying the result to the experimental data. The inverse method might provide a more accurate means of inferring cluster number and other intrinsic properties from the experimental data, as well as obtaining tighter confidence limits on those estimates. This would be anticipated because the training distribution would be made much more similar to the experimental distribution by design, and thus be better suited to model the experimental data for purposes of regression. The caveat is that the resultant regression model might not be as generalizable to other datasets as the current method that uses random distributions for training.

To conclude, more research is needed to explore the capabilities and limitations of this blind method for determining intrinsic clustering properties in multidimensional datasets. However, the results of this work establish that hierarchical linkage regression may be a useful technique for investigating datasets in which there is an unknown number of groupings embedded within the data, and therefore has the potential to become a promising tool for data mining and analysis, with foreseeable application in finance, marketing, industrial automation, quality control, genetic analysis and drug discovery, to name a few.

## Supporting information

S1 Table Model comparison for single cluster number evaluation—Summary of cluster number estimate statistics. (DOCX)

S2 Table Model recall comparison for single cluster number evaluation—Normally-distributed random data. (DOCX)

S3 Table Model recall comparison for single cluster number evaluation—Image data. (DOCX)

S4 Table Model recall comparison for single cluster number evaluation—Text data. (DOCX)

S5 Table Mean F1 score model comparison for multi-cluster number evaluation. (DOCX)
