# Supplementary material for: Blind method for discovering number of clusters in multidimensional datasets by regression on linkage hierarchies generated from random data
Source: PLoS One. 2020 Jan 23;15(1):e0227788. doi: 10.1371/journal.pone.0227788 (PMC6977736; doi:10.1371/journal.pone.0227788)
Supplement: S1 Table — (DOCX) [file pone.0227788.s001.docx]

**S1 Table. Model comparison for single cluster number evaluation – summary of cluster number estimate statistics.**

|  | | **CH** | | **DB** | | **S** | | **G** | | **AP** | **DBSN** | **OPTICS** | **HLR** |
| --- | --- | --- | --- | --- | --- | --- | --- | --- | --- | --- | --- | --- | --- |
|  |  | **-L** | **-K** | **-L** | **-K** | **-L** | **-K** | **-L** | **-K** |  |  |  |  |
| **Ideal** | **mean** | 13.4 | | 14.9 | | 14.9 | | 18.8 | | 16.8 | 14.0 | 12.6 | 15.6 |
|  | **median** | 15.0 | | 15.0 | | 15.0 | | 16.0 | | 16.0 | 15.0 | 15.0 | 15.6 |
|  | **std** | 3.6 | | 0.5 | | 0.5 | | 5.0 | | 2.1 | 2.9 | 4.8 | 1.7 |
| **Image** | **mean** | 5.2 | 2.7 | 5.3 | 8.7 | 3.0 | 4.3 | 29.9 | 29.4 | 2.4 | 1.1 | 10.1 | 14.3 |
|  | **median** | 3.0 | 2.0 | 2.0 | 3.0 | 2.0 | 2.0 | 30.0 | 30.0 | 2.0 | 1.0 | 10.0 | 14.5 |
|  | **std** | 5.2 | 1.2 | 7.3 | 9.6 | 3.4 | 5.7 | 0.3 | 0.8 | 0.9 | 0.2 | 2.5 | 1.9 |
| **Text** | **mean** | 3.6 | 2.6 | 7.3 | 9.3 | 13.4 | 10.9 | 28.7 | 0.0 | 22.0 | 2.5 | 8.2 | 13.6 |
|  | **median** | 2.0 | 2.0 | 7.0 | 10.0 | 12.0 | 11.0 | 29.0 | 0.0 | 22.0 | 2.0 | 8.0 | 14.0 |
|  | **std** | 2.5 | 1.2 | 3.5 | 2.5 | 7.6 | 2.7 | 0.5 | 0.0 | 3.1 | 1.4 | 3.1 | 3.2 |

Mean, median and standard deviation of cluster number estimates for ground-truth ($\hat{y}$ = 15) for ideal (normally-distributed random) data, texture image data and Wiki text data. Legend is as in Fig 8.
