# Supplementary material for: Blind method for discovering number of clusters in multidimensional datasets by regression on linkage hierarchies generated from random data
Source: PLoS One. 2020 Jan 23;15(1):e0227788. doi: 10.1371/journal.pone.0227788 (PMC6977736; doi:10.1371/journal.pone.0227788)
Supplement: S2 Table — (DOCX) [file pone.0227788.s002.docx]

**S2 Table. Model recall comparison for single cluster number evaluation – normally-distributed random data**.

| $\Delta$ | **CH** | **DB** | **S** | **G** | **AP** | **DBSN** | **OPTICS** | **HLR** |
| --- | --- | --- | --- | --- | --- | --- | --- | --- |
| **0** | 0.73 | 0.89 | 0.92 | 0.37 | 0.46 | 0.74 | 0.71 | 0.26 |
| **1** | 0.79 | 0.96 | 0.96 | 0.58 | 0.55 | 0.8 | 0.77 | 0.62 |
| **2** | 0.84 | 1 | 0.99 | 0.64 | 0.65 | 0.86 | 0.78 | 0.8 |
| **3** | 0.85 | 1 | 1 | 0.69 | 0.73 | 0.9 | 0.78 | 0.95 |
| **4** | 0.85 | 1 | 1 | 0.7 | 0.84 | 0.91 | 0.78 | 0.99 |
| **5** | 0.89 | 1 | 1 | 0.7 | 0.95 | 0.92 | 0.8 | 0.99 |

Recall values for estimates within $\Delta$ clusters of ground-truth ($\hat{y}$ = 15). Legend is as in Fig 9.
