# Supplementary material for: Blind method for discovering number of clusters in multidimensional datasets by regression on linkage hierarchies generated from random data
Source: PLoS One. 2020 Jan 23;15(1):e0227788. doi: 10.1371/journal.pone.0227788 (PMC6977736; doi:10.1371/journal.pone.0227788)
Supplement: S3 Table — (DOCX) [file pone.0227788.s003.docx]

**S3 Table. Model recall comparison for single cluster number evaluation – image data**.

| $\Delta$ | **CH-L** | **CH-K** | **DB-L** | **DB-K** | **S-L** | **S-K** | **G-L** | **G-K** | **AP** | **DBSN** | **OPTICS** | **HLR** |
| --- | --- | --- | --- | --- | --- | --- | --- | --- | --- | --- | --- | --- |
| **0** | 0.01 | 0 | 0 | 0.02 | 0 | 0.01 | 0 | 0 | 0 | 0 | 0 | 0.23 |
| **1** | 0.01 | 0 | 0 | 0.04 | 0 | 0.01 | 0 | 0 | 0 | 0 | 0.07 | 0.55 |
| **2** | 0.02 | 0 | 0.01 | 0.04 | 0.01 | 0.01 | 0 | 0 | 0 | 0 | 0.12 | 0.79 |
| **3** | 0.03 | 0 | 0.02 | 0.06 | 0.02 | 0.04 | 0 | 0 | 0 | 0 | 0.32 | 0.9 |
| **4** | 0.05 | 0 | 0.04 | 0.11 | 0.02 | 0.04 | 0 | 0 | 0 | 0 | 0.44 | 0.99 |
| **5** | 0.08 | 0 | 0.07 | 0.13 | 0.03 | 0.05 | 0 | 0 | 0 | 0 | 0.58 | 1 |

Recall values for estimates within $\Delta$ clusters of ground-truth ($\hat{y}$ = 15). Legend is as in Fig 9.
