# Supplementary material for: Blind method for discovering number of clusters in multidimensional datasets by regression on linkage hierarchies generated from random data
Source: PLoS One. 2020 Jan 23;15(1):e0227788. doi: 10.1371/journal.pone.0227788 (PMC6977736; doi:10.1371/journal.pone.0227788)
Supplement: S4 Table — (DOCX) [file pone.0227788.s004.docx]

**S4 Table. Model recall comparison for single cluster number evaluation – text data**.

| $\Delta$ | **CH-L** | **CH-K** | **DB-L** | **DB-K** | **S-L** | **S-K** | **G-L** | **G-K** | **AP** | **DBSN** | **OPTICS** | **HLR** |
| --- | --- | --- | --- | --- | --- | --- | --- | --- | --- | --- | --- | --- |
| **0** | 0 | 0 | 0 | 0 | 0.03 | 0.03 | 0 | 0 | 0.01 | 0 | 0.01 | 0.12 |
| **1** | 0 | 0 | 0.02 | 0.04 | 0.19 | 0.16 | 0 | 0 | 0.04 | 0 | 0.05 | 0.41 |
| **2** | 0 | 0 | 0.02 | 0.05 | 0.22 | 0.28 | 0 | 0 | 0.11 | 0 | 0.1 | 0.74 |
| **3** | 0.02 | 0 | 0.09 | 0.18 | 0.32 | 0.45 | 0 | 0 | 0.14 | 0 | 0.18 | 0.8 |
| **4** | 0.02 | 0 | 0.19 | 0.29 | 0.44 | 0.59 | 0 | 0 | 0.21 | 0 | 0.22 | 0.89 |
| **5** | 0.03 | 0 | 0.28 | 0.52 | 0.48 | 0.69 | 0 | 0 | 0.35 | 0 | 0.3 | 0.92 |

Recall values for estimates within $\Delta$ clusters of ground-truth ($\hat{y}$ = 15). Legend is as in Fig 9.
