# Supplementary material for: Blind method for discovering number of clusters in multidimensional datasets by regression on linkage hierarchies generated from random data
Source: PLoS One. 2020 Jan 23;15(1):e0227788. doi: 10.1371/journal.pone.0227788 (PMC6977736; doi:10.1371/journal.pone.0227788)
Supplement: S5 Table — (DOCX) [file pone.0227788.s005.docx]

**S5 Table. Mean F1 score model comparison for multi-cluster number evaluation**

|  | $\Delta$ | **Davies-Bouldin** | | **Silhouette** | | **OPTICS** | | **HLR** | |
| --- | --- | --- | --- | --- | --- | --- | --- | --- | --- |
|  |  | $\bar{\mathbf{F}\boldsymbol{1}}$ | **±std** | $\bar{\mathbf{F}\boldsymbol{1}}$ | **±std** | $\bar{\mathbf{F}\boldsymbol{1}}$ | **±std** | $\bar{\mathbf{F}\boldsymbol{1}}$ | **±std** |
| **Ideal** | **0** | 0.52 | 0.32 | 0.84 | 0.24 | 0.37 | 0.33 | 0.45 | 0.16 |
|  | **1** | 0.73 | 0.17 | 0.94 | 0.04 | 0.48 | 0.30 | 0.76 | 0.10 |
|  | **2** | 0.83 | 0.08 | 0.96 | 0.02 | 0.59 | 0.24 | 0.86 | 0.05 |
|  | **3** | 0.88 | 0.05 | 0.97 | 0.02 | 0.68 | 0.20 | 0.91 | 0.03 |
|  | **4** | 0.91 | 0.04 | 0.97 | 0.02 | 0.76 | 0.14 | 0.93 | 0.02 |
|  | **5** | 0.92 | 0.04 | 0.97 | 0.01 | 0.81 | 0.09 | 0.95 | 0.01 |
| **Image** | **0** | 0.00 | 0.01 | 0.01 | 0.02 | 0.08 | 0.10 | 0.26 | 0.12 |
|  | **1** | 0.02 | 0.02 | 0.06 | 0.08 | 0.21 | 0.21 | 0.55 | 0.12 |
|  | **2** | 0.04 | 0.03 | 0.11 | 0.14 | 0.31 | 0.26 | 0.71 | 0.08 |
|  | **3** | 0.06 | 0.04 | 0.18 | 0.20 | 0.39 | 0.28 | 0.79 | 0.05 |
|  | **4** | 0.09 | 0.05 | 0.25 | 0.26 | 0.47 | 0.31 | 0.84 | 0.03 |
|  | **5** | 0.12 | 0.06 | 0.34 | 0.31 | 0.56 | 0.32 | 0.87 | 0.02 |
| **Text** | **0** | 0.20 | 0.18 | 0.18 | 0.14 | 0.04 | 0.08 | 0.20 | 0.15 |
|  | **1** | 0.38 | 0.18 | 0.39 | 0.15 | 0.09 | 0.14 | 0.51 | 0.13 |
|  | **2** | 0.52 | 0.15 | 0.52 | 0.13 | 0.15 | 0.20 | 0.69 | 0.08 |
|  | **3** | 0.62 | 0.13 | 0.61 | 0.12 | 0.22 | 0.26 | 0.78 | 0.04 |
|  | **4** | 0.71 | 0.11 | 0.67 | 0.10 | 0.29 | 0.32 | 0.83 | 0.02 |
|  | **5** | 0.77 | 0.10 | 0.73 | 0.09 | 0.37 | 0.36 | 0.87 | 0.02 |

Mean F1 score values and their standard deviations for estimates within $\Delta$ clusters of ground-truth ($\hat{y}$ = 1 to 15) for ideal (normally-distributed random) data, texture image data and Wiki text data.
